# Control of Thruster-Assisted, Bipedal Legged Locomotion of the Harpy Robot

**DOI:** 10.3389/frobt.2021.770514

**Published:** 2021-12-23

**Authors:** Pravin Dangol, Eric Sihite, Alireza Ramezani

**Affiliations:** Silicon Synapse Lab., Department of Electrical and Computer Engineering, Northeastern University, Boston, MA, United States

**Keywords:** legged robots, dynamics, modeling, control, mechatronics, automation

## Abstract

Fast constraint satisfaction, frontal dynamics stabilization, and avoiding fallovers in dynamic, bipedal walkers can be pretty challenging. The challenges include underactuation, vulnerability to external perturbations, and high computational complexity that arise when accounting for the system full-dynamics and environmental interactions. In this work, we study the potential roles of thrusters in addressing some of these locomotion challenges in bipedal robotics. We will introduce a thruster-assisted bipedal robot called *Harpy*. We will capitalize on Harpy’s unique design to propose an optimization-free approach to satisfy gait feasibility conditions. In this thruster-assisted legged locomotion, the reference trajectories can be manipulated to fulfill constraints brought on by ground contact and those prescribed for states and inputs. Unintended changes to the trajectories, especially those optimized to produce periodic orbits, can adversely affect gait stability and hybrid invariance. We will show our approach can still guarantee stability and hybrid invariance of the gaits by employing the thrusters in Harpy. We will also show that the thrusters can be leveraged to robustify the gaits by dodging fallovers or jumping over large obstacles.

## 1 Introduction

Raibert’s hopping robots Raibert et al. (1984), and Boston Dynamic’s BigDog Raibert et al. (2008) are amongst the most successful examples of legged robots, as they can hop or trot robustly even in the presence of significant unplanned disturbances. Other than these successful examples, many bipedal and anthropomorphic robots have also been introduced. Boston Dynamics’ dynamic humanoid, ATLAS, has pushed the limits of dynamic legged locomotion with its 28 hydraulically actuated joints. This robot has showcased impressive mobility feats, including jumping over obstacles and dynamic flip-turns. Unfortunately, there are no publications laying out the details of Boston Dynamics works. Therefore, little is known about the controllers used in ATLAS.

Another successful example is Cassie. Agility Robotics developed this bipedal robot based on an earlier prototype called ATRIAS led by Oregon State University Apgar et al. (2018). The biped can negotiate unstructured environments such as ramps and stairs inside buildings robustly and efficiently. With 20 Degrees of Freedom (DOF) and ten actuators, Cassie is a hard-to-control floating base possessing 6 Degrees of Under-actuation (DOU). With a smaller number of DOU than Cassie and ATRIAS and a restricted frontal dynamics, Michigan’s MABEL possesses pogo-stick-style feet, compliant legs, and an anthropomorphic morphology. This robot has shown stable and natural running gaits similar to humans Sreenath et al. (2011). Completely blind and relied on no visual feedback, MABEL has showcased stable gaits, which involve traversing along rough terrains even when no information about the bumps’ whereabouts in its path is available Park et al. (2012).

Fully actuated systems such as Valkyrie, ASIMO, Mahru, and Yobotics-IHMC have shown impressive performance similar to the above examples. NASA’s Johnson Space Center led a team of partners from academia and industry and developed NASA’s first bipedal humanoid, Valkyrie Paine et al. (2015). Valkyrie has showcased the successful completion of sophisticated human-style tasks in the DARPA Robotic Challenge (DRC). Other examples such as Honda’s ASIMO Hirose and Ogawa (2006) and Samsung’s Mahru III Kwon et al. (2007) have demonstrated capabilities such as quasi-static walking, running, dancing, and climbing stairs inside buildings. Or, Yobotics-IHMC lower body, humanoid biped has shown recovery from severe pushes Pratt et al. (2009).

Despite all of these accomplishments, state-of-the-art bipedal robots are prone to fall-over and cannot negotiate highly rough terrains. Even humans known for their natural, efficient, and robust locomotion feats cannot recover from unpredictable situations such as severe external pushes, scuffing, or slippage on icy surfaces.

**FIGURE 1:**
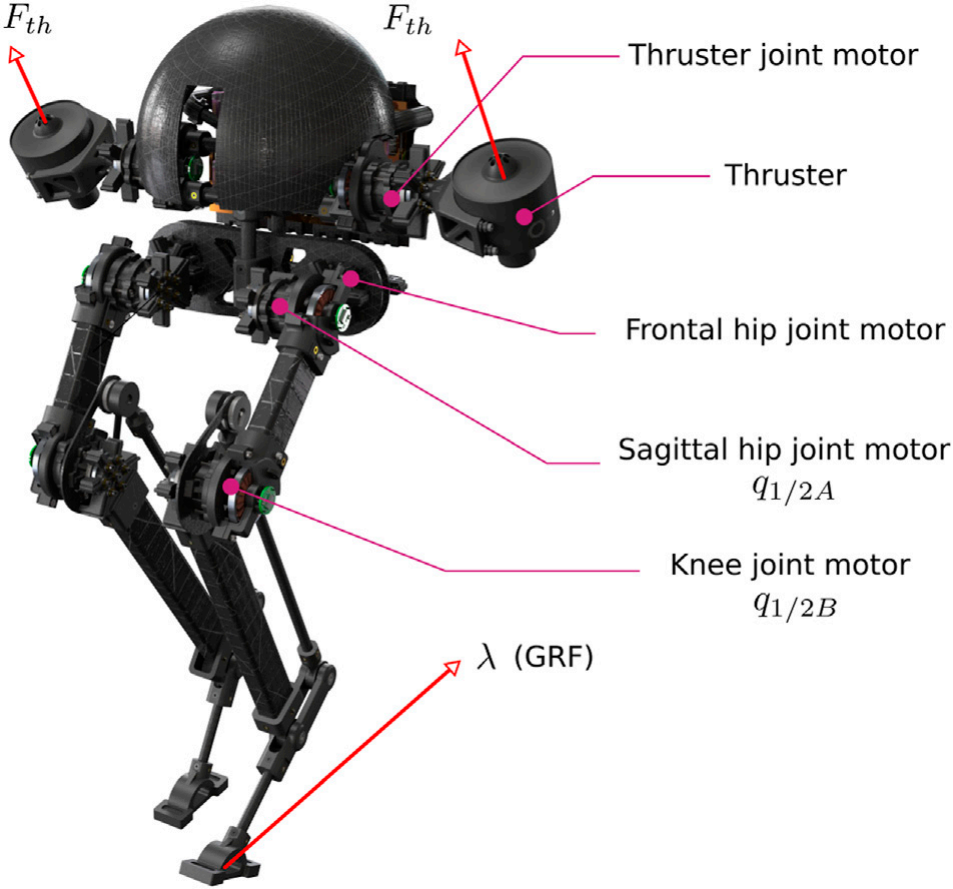
Illustration of *Harpy’s* CAD model. Harpy is a bipedal robot with a pair of thrusters and is being developed by the authors to study robust, efficient and agile thruster-assisted legged locomotion.

Our main goal is to enhance bipedal systems’ robustness through distributed arrays of thrusters and nonlinear control. This paper will report our recent efforts in dynamic modeling and designing closed-loop feedback for the thruster-assisted locomotion of a legged robot called Harpy, which is shown in Figure 1. Currently, Harpy’s hardware is being developed at Northeastern University (NU). This bipedal robot is equipped with a total number of eight custom-made joint actuators, a pair of coaxial thrusters fixed to Harpy’s torso, and a light body structure fabricated out of reinforced carbon fibers.

The overarching goal is to integrate aerial and legged systems’ capabilities in a single platform. Aerial robots possess fast mobility mainly because they can fly over obstacles. This property of aerial systems can be highly suitable for applications such as automated package delivery or monitoring and inspection from vantage points. While legged robots may have difficulty negotiating extremely bumpy terrains, e.g., semi-collapsed buildings in the aftermath of an earthquake, these systems maintain a superior energetic efficiency of locomotion compared to aerial drones.

Unlike aerial robots, legged systems can safely operate near humans and animals and are very suitable for many applications such as search and rescue inside buildings, digital agriculture, monitoring livestock for disease symptoms, assisting workers in construction zones, etc. In all of these applications, the fast-rotating blades in rotary-wing robots can result in severe laceration injuries. As a result, there are strict rules on aerial systems’ operation near humans.

From a control design perspective, thruster-assisted legged locomotion has not been explored previously. In this work, we are particularly interested in the closed-loop feedback design implications of thrusters, and our objective is to apply thrusters to achieve two distinguished goals. We will briefly discuss these goals below.

### 1.1 Disturbance Rejection in the Sagittal and Frontal Planes of Locomotion

One objective is to use the thrusters and robustify the gaits. Bipedal platforms are incredibly vulnerable to disturbance and fall-over. Environment and model disturbances are common contributors to these systems’ tip-over instability (rotational and postural) Goswami and Kallem (2004), which can lead to complete loss or severe damages to the system. Because of the inherent complexity of disturbance rejection, a handful of mainstream strategies have been successfully applied to solve such a vulnerability Gubina et al. (1974); Pratt and Tedrake (2006); Kajita and Tani (1991); Hill and Fahimi (2015); Mandava and Vundavilli (2018); Song et al. (2018); Arcos-Legarda et al. (2019). None of these strategies have been able to mitigate tip-over scenarios entirely.

Rotational stability can be achieved if the external forces and moments acting on the system yield zero centroidal moments, i.e., the system’s overall angular momentum is conserved Vukobratović and Stepanenko (1972). External forces and moments may arise from various sources, such as gravity, immature ground contacts, and unexpected external disturbances. To retain rotational stability the orientation of the Ground Reaction Force (GRF) should be regulated relative to the Center of Mass (COM).

Bipedal robots cannot directly control the magnitude and orientation of the GRF because of the unilaterality of the contact forces. However, they can modulate the GRF indirectly through the nonlinear couplings in these systems. The unilaterality of GRF yields a considerably limited tip-over recovering and disturbance rejection capability. For instance, when the extent of the disturbance force is moderately small, e.g., a gentle push, small body movements can be leveraged to retain the robot’s rotational balance.

Upper body movements can be applied to induce restoring moments at the hips and ankles by transferring angular moments. Traditional forward and inverse kinematics frameworks that place virtual components such as dampers and springs in strategic locations in the robot have been successfully applied to describe these robot-environment interactions Pratt et al. (2001). Still, their use is limited to minor disturbances.

Other widely applied methods based on Center of Pressure (COP) control are limiting. Only limited to quasi-steady gaits, these frameworks locate and regulate the COP of a bipedal robot within the support polygon through various strategies, e.g., applying ankle torques, to oppose the tip-over moment. That said, the effectiveness of these methods is limited. The movement of the COP position along the feet, which is proportional to the torque generated at the ankle, can end to the boundaries of the support polygon, and tip-over can occur. As a result, the support polygon’s size is often interpreted as the decision (stability) margin in the state space of the COM. The position of the COM relative to the support polygon is constantly monitored to decide whether the COP-based controller is enough to retain stability or not Pratt et al. (2006).

More aggressive disturbance rejection mechanisms such as a flywheel effect allow for additional momentum contribution required to retain a bipedal robot’s stability against more prominent external disturbances, e.g., a hard impulsive push force applied to the system Stephens (2007). However, the flywheel method’s effectiveness can be limited mainly because significant resisting torques must be applied to the joints to terminate the flywheel effect after the disturbance is attenuated, which can yield other issues such as the violation of contact forces at leg-end points. Larger disturbances can be handled by taking a few compensating steps, just like humans retaining their balance under sudden pushes. While this approach is very promising, its effectiveness is highly affected by other limitations in the step kinematics and impact dynamics (the topology of terrain).

### 1.2 Dynamic Walking and Performance Satisfaction

Another objective is to explore performance satisfaction paradigms superior to existing methods. We will briefly explain this objective here.

Dynamic bipedal walkers possess small support polygons. Small stability margin and underactuation at the contact points result in severe challenges to achieve energetically efficient and robust gaits. These two antagonistic properties are often achieved at the cost of sacrificing one another, and satisfying efficiency and robustness at the same time is still an open problem. Examples such as the robust locomotion feats of ATLAS robot achieved through the application of energy-hungry hydraulic actuators and the efficient gaits of Cornell’s passive walker with a minimum number of electric actuators are two notable examples that fall on the two opposite ends of the performance spectrum.

The problem of simultaneously providing asymptotic stability, optimizing desired performance indices, and satisfying gait constraints in legged systems has been studied extensively Westervelt and Grizzle (2007); Galloway et al. (2015); Dai and Tedrake (2016); Feng et al. (2014). However, the challenges remain, and there are severe limits with existing methods. For instance, model-based methods such as Hybrid Zero Dynamics (HZD), which have offered rigorous ways to assign attributes such as a minimized total cost of transport and robustness in an off-line fashion, are restrictive for dynamic scenarios involving joint trajectory re-planning. Or, in work by, despite increased cost-of-transport optimization accuracy without additional computational time using a variable-time-interval trajectory optimization method, obtaining optimal solutions remain restricted to simple models and slow gaits.

To better understand existing options, we categorize performance satisfaction in dynamic systems – regardless of being a legged or non-legged robot (e.g., robot manipulators) – into three broad categories. Namely: 1) trajectory optimization (TO), 2) optimization-based (nonlinear) controls, and 3) reference trajectory manipulation.

TO problems’ goal is to generate optimal trajectories that satisfies constraints on states, inputs, and GRF while ensuring that the trajectories lead to stable walking gaits. TO problems for the legged robot are challenging to solve due to their nonlinear dynamics, high degrees of freedom and the system’s hybrid nature brought on by ground impact. Previous works such as Posa et al. (2014); Hereid et al. (2016); Medeiros et al. (2020) have proposed methods to transcribe this as a Nonlinear Programming (NLP) problem through direct collocation methods where polynomial splines are used to approximate the continuous dynamics and thus reducing computational complexity without needing to account for the actual dynamics. Other works such as Hereid et al. (2015) have instead proposed utilizing multiple shooting methods to break the original problem down into smaller steps without approximations.

In both cases, however, the system’s dynamics need to be considered along with contact dynamics to generate the trajectories. These works Pardo et al. (2017); Xin et al. (2020), to dodge the need to define ground contact dynamics explicitly, have employed null space projection methods. Meaning, zero acceleration constraints are enforced on feet ends in Hereid et al. (2016). The issue remains, and these methods are extremely computationally expensive and cannot be implemented in real-time, taking a few minutes to solve the TO problem. For works that use reduced-order models such as the centroidal dynamics or utilize Zero Moment Point (ZMP) based methods as in Pratt et al. (2006); Winkler et al. (2015), experimental results of online optimization are available. These results are restricted to pseudo-static gaits rather than dynamic ones.

In the second category (i.e., optimization-based control schemes), the goal is to compute constraint-aware feedback stabilizing control loops. This goal is most commonly achieved through a predictive framework, usually by creating a linearized model over a finite-time horizon. For instance, in Bledt et al. (2018); Fahmi et al. (2019); Angelini et al. (2019), in a hierarchical framework, reduced-order models around the COM are used to generate reference acceleration for the low-level tracking controller. The downfall of these options is the need to linearize and simplify the robot’s underlying dynamics to make it feasible in real-time, and as a result, not all constraints can be considered. In Hutter et al. (2014); Xin et al. (2020); Galloway et al. (2015); Nguyen and Sreenath (2016), the desired control inputs are computed taking into account the full-dynamics, and then optimization is carried out on a simplified least square or QP problem for tractability.

A different approach, which is the focus of the third category in our list, is to remove optimization from the control strategy and instead modify the reference trajectories to obey desired constraints. This idea was popularized through Reference Governors (RG) Kapasouris et al. (1988), where an efficient online optimization method is employed. The core to this idea is that the reference trajectory that the controller must follow can be manipulated while keeping it close to the original trajectory in the event that boundaries created by constraints are to be violated. Since its inception, this idea has spawned many variations Kolmanovsky et al. (2012); Garone et al. (2017) including an optimization-free approach known as Explicit Reference Governor (ERG) Nicotra and Garone (2015). Besides the possibility of utilizing an optimization-free form, the other major advantage with RG is that it acts as an add-on scheme to an existing controller without the need for any modification on the control scheme. However, these methods have only proven useful in relatively simple nonlinear systems as their applications to high-dimensional nonlinear problems are nearly impossible.

Other emerging paradigms such as Approximate Dynamic Programming (ADP), reinforcement learning, decoupled approaches to design control for nonlinear stochastic systems, etc., can potentially remedy the challenges in the future. For instance, employed a learning method to understand the stability and robustness of stability against the external disturbances of a passive biped walker. They used a multi-objective, multi-modal particle swarm optimization algorithm to find stable initial conditions for their biped walker model. These approaches are currently far from providing any practical solutions to the problem of performance satisfaction in dynamic bipedal walkers. They are shown to be only effective on simpler practical robots, mainly those that can only demonstrate quasi-static gaits.

## 2 Contributions and the Summary of the Proposed Framework

While gait design for complex, multi-DOF legged robots based on full or reduced-order models has been addressed extensively, optimization-free gait re-design in a reactive fashion and within gait strides are often considered only for quasi-static walkers. In dynamic walkers, such reactive gait adjustments take very complex forms involving optimization problems to ensure gait feasibility conditions.

One primary reason that within-strides gait adjustment in dynamic walkers involves optimization is that these bipeds possess small support polygons that leave small to no stability margins and make gait adjustment very intricate. With this observation and to combat the challenges associated with dynamic walking, our primary objective is to apply novel thrust-vectoring-based control actions in bipedal legged locomotion. Despite their potential merits, as we discussed above, the application of these thrusters has remained unexplored and existing studies, are limited to only flight control and not thruster-assisted legged locomotion.

Specifically speaking, the contribution of this work achieved due to thrusters’ presence in our robot is four-pronged.

• The first contribution of this work is that it employs thruster actions during the gait cycles for several vital reasons. These objectives include securing frontal dynamics stability, avoiding sagittal plane fallovers, and making aggressive jumps over obstacles. Previously, to achieve these goals, bipedal-legged robots entirely relied on indirect methods such as posture control (at stance phase) and rules of conservation of angular momentum (at flight phase during running) to avoid these fallover scenarios.

• This work introduces a decoupled view towards satisfying two antagonistic properties of efficiency and robustness. Meaning it shows that one can consider part of the gait cycle for performance satisfaction and the rest of the step period for gait robustification. The application of this decoupled view towards performance satisfaction is impossible in conventional bipeds. As a result, the decoupled idea remained unexplored. A major consequence of applying the decoupled view is that it allows the application of simpler computational algorithms for joint trajectory planning (or re-planning), control, and GRF constraint satisfaction, which constitute the next contribution of this work.

• Introducing an efficient controller, which secures the feasibility of the gaits and constraints, is the third contribution of this work. This work presents a systematic approach based on reference governors, which are relied on provable Lyapunov stability properties, to minimize computational overhead. This approach permits performing motion planning in the state-space of the Zero Dynamics (ZD) of our bipedal robot, yielding a significantly lower cost of computation than widely used optimization-based methods applied on full- or reduced-order dynamical models. To comprehensively describe this method, we will extend our previous works Dangol et al. (2020); Dangol and Ramezani (2020) by conjointly employing state-space motion planning tools and celebrated bipedal robot control design frameworks such as the HZD method.

We take a simulation-based approach to validate our method. We will use the simulators developed for our Harpy platform, capitalize on the thrusters’ action to resolve and re-design Harpy’s gait parameters during its Single Support (SS) phase. The SS phase is the most extended phase in Harpy’s gait cycle. Then, we will assume well-tuned supervisory controllers within our reference governor model and focus on fine-tuning Harpy’s joints’ desired trajectories to satisfy gait feasibility constraints, including saturating controls and retaining feasible contact forces.

• Finally, this work shows that achieving hybrid invariance in the face of significant external perturbations, in a finite-time fashion, using thrust vectoring is possible. Here, hybrid invariance is violated for an important reason. Since we devise intermediary filters based on RGs, there can be a mismatch between the robot’s states at the gaits’ boundaries. Put differently, Harpy’s gait modifications and impact events can lead to severe deviations from its nominal periodic orbits. In this work, we demonstrate that owing to Harpy’s thrusters hybrid invariance will be achieved by either applying the thrusters throughout the whole gait cycle or employing them intermittently.

This work is organized as follows. In Section 3, we will briefly introduce our platform, Harpy. We will avoid reporting hardware-related matters in this work as they are beyond the scope of this publication. Harpy’s hardware will be reported in subsequent publications. In Section 4, Harpy’s planar dynamics, which consist of continuous and impact models, will be derived. The robot’s SS phase will be modeled as a Reduced-Order Model (ROM) following standard conventions and assumptions found in textbooks Westervelt and Grizzle (2007). Then, a two-point DS model followed by an impact map for a non-instantaneous phase will be obtained. Hybrid invariance will be achieved using a Nonlinear Model Predictive Control (NMPC) scheme during the DS phase. Gait design based on HZD method and satisfying performance constraints based on RG will be explained in detail. Last, our simulation results will be discussed in Section 6 as we will report the performance of the proposed approach.

## 3 Quick Overview of Harpy Platform

This section will discuss Harpy’s physical properties and elaborate on our philosophy regarding the design. Also, we will briefly discuss the weight considerations and mass allocation and their impact on modeling.

### 3.1 Physical Properties

The system, shown in Figure 1, weighs roughly 4 lbs. It is 2.4 ft tall. The legs are composed of a parallelogram mechanism. With a highly integrated and unified actuation and sensing applied in the design of Harpy, which will be explained below, minimum use of metal components, including fasteners, housings, support structures, etc., is achieved. Parts are designed for modularity, energy efficiency, and low weight. It is worth noting that high energy-to-weight ratios are critical characteristics of birds which are our design role models. Birds are capable of showcasing legged and aerial locomotion to perfection.

### 3.2 Upper Body Design

**FIGURE 2:**
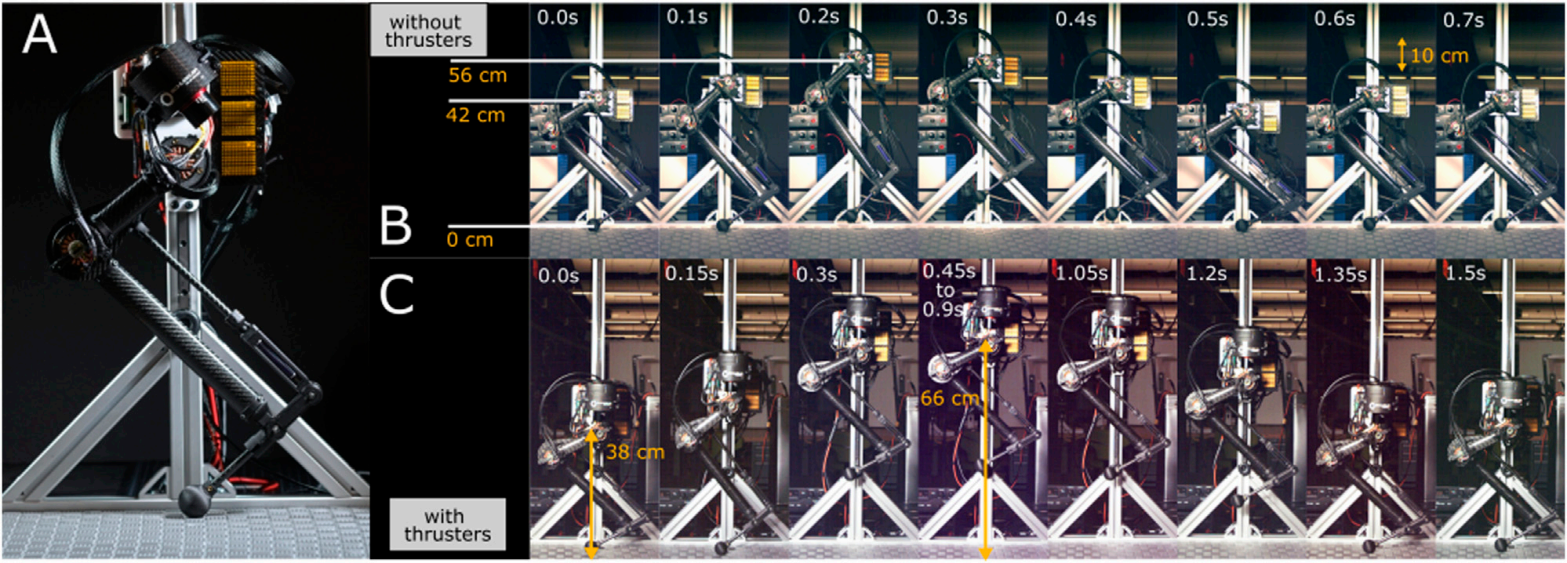
**(A)** Illustrates the current status of the ongoing Harpy hardware developments at Northeastern University. **(B,C)** Show successful mono-pedal jumping tests with and without thrusters. A gantry supports the half-finished prototype in these tests.

The torso, shown in Figure 2, hosts two brush-less DC actuators with harmonic drives to increase the output torque. These actuators are used to move the legs in the frontal plane of motion. Two co-axial thrusters are attached to a separate actuator and can move relative to the torso in the sagittal plane of locomotion. We will use the thrusters for the following purposes: 1) to directly regulate the ground contact forces, 2) to recover the system when incidental tip-overs occur, 3) to generate the lift force required for jumping over obstacles, 4) to stabilize the frontal dynamics and 5) to secure hybrid invariance. Additionally, the torso encapsulates power electronics, including the amplifiers for actuators and thrusters, sensors, communication nodes, and a computer for online processing and closed-loop feedback.

The robot is underactuated when the thrusters are off and can be overactuated when active. Indeed, when Harpy is unconstrained, i.e., it is not in contact with the environment, it possesses 14 DOF. Six DOF are associated with the translation and orientation of the robot. There are two DOF at the locations where the hips are attached to the torso.

### 3.3 Payload Reduction and Added Mass Challenge

The mechanical design and actuation approach applied in Harpy is different from existing platforms. Instead of the generous incorporation of metal components, it has been tried to rely on composite material, fiber-inlay additive manufacturing, and embedding methods. The design approach based on embedding all of the components within the composite structure of the robot has been applied to achieve an ultra-light robot. Gaining a minimized total weight is essential in Harpy’s design. Harpy can be looked upon as a self-manipulating system therefore, large payloads can lead to the need for stronger actuators or thrusters. These actuators are often heavier, and consequently, extra mass can be introduced in the design.

### 3.4 Spring-Loaded Inverted Pendulum Model Design Considerations

The actuators and Harpy’s structure are designed to deliver a large range of motion on all DOF. Low-inertia limbs are considered to capture Spring-Loaded Inverted Pendulum (SLIP) model characteristics. Each leg, shown in Figure 1, possesses eight DOF and after considering the mechanical couplings three DOF remain. Back-driveable, harmonic drive actuators replace extra torque or force sensors or series compliance which can help reduce the overall mass of Harpy’s legs.

Low limb inertia and low reflected actuator inertia make the robot capable of extremely fast leg-swings. Besides, the hip and leg actuators are located so that their axes of rotation intersect at the hip joint. This design consideration helps reduce the moment of inertia in the frontal plane. The inherent compliance in the legs, particularly in the 4-bar linkage, can potentially reduce mechanical bandwidth in the legs. This property can affect foot placement performance. In a recently completed platform that uses a similar leg mechanism called Husky, fast and precise foot placement was achieved, supporting the feasibility of Harpy’s leg design.

## 4 Harpy Modeling

**FIGURE 3:**
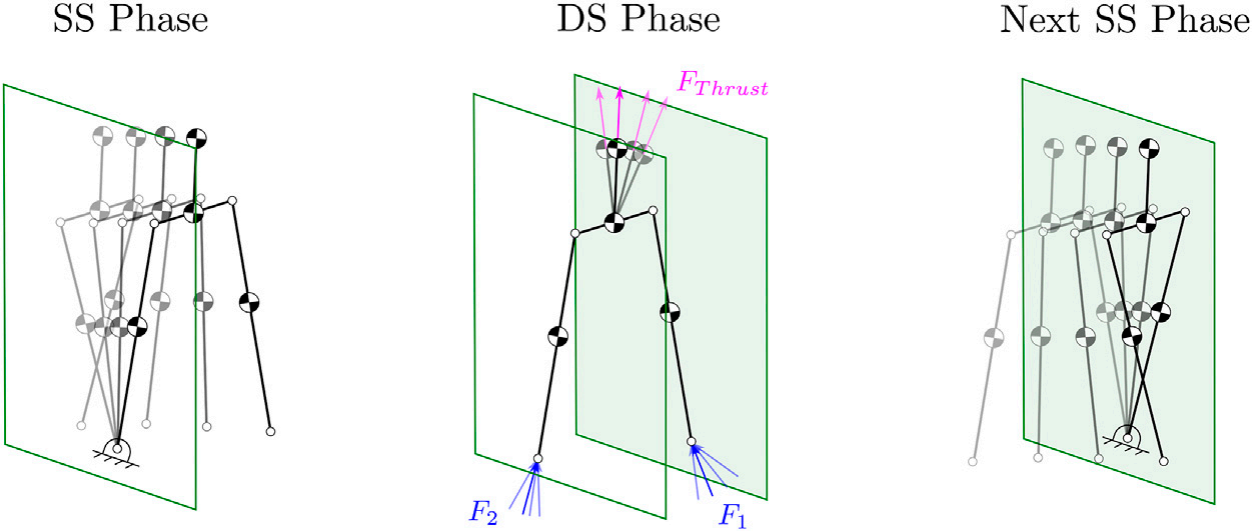
Illustration of the SS and DS phases. Also, shows the thrusters utilized to regulate the GRF.

This section will briefly outline Harpy configuration space, underactuated and actuated coordinates, full-model, and ROMs. We will break down the gait cycle into two distinct phases: 1) an SS phase during which only one leg, called the stance leg, is in constant contact with the ground and 2) a DS phase during which both legs are fixated to the ground for a very short period. The transitions, including SS to DS and DS to SS, will be marked by the swing leg touch-down and lift-off, respectively as shown in Figure 3.

Assuming point-foot walking, we will design predefined periodic gaits for the system following the well-established design framework of HZD as discussed in Westervelt et al. (2003). We will avoid discussing gait parameterization, design, and ZD derivations as the procedure has been exhaustively reported in the literature.

### 4.1 Configuration Space and Control Inputs

We assume a body coordinate frame attached to the torso COM with the x- and *z*-axis pointing forwards and upwards, respectively. During the SS phase, Harpy’s full-model possesses 14 DOF. The joint angles include roll (*q*_*r*_), pitch (*q*_*p*_), yaw (*q*_*y*_), thigh (*q*_*iThigh*_), knee (*q*_*iKnee*_), hip (*q*_*iHip*_), thruster (*q*_*iThrust*_) angles for $i\in \left\{1\left(stance\;leg\right),2\left(swing\;leg\right)\right\}$ and stance leg-end positions $\left(p_{1}={\left[p_{tx},p_{ty},p_{n}\right]}^{⊤}\right)$ . All of these variables are stacked inside the vector $q_{s}={\left[q_{y},q_{p},q_{r},q_{iThigh},q_{iKnee},q_{iHip},q_{iThrust},p_{1}^{⊤}\right]}^{⊤}$ to form the states vector $x_{s}={\left[q_{s}^{⊤},{\dot{q}}_{s}^{⊤}\right]}^{⊤}$ .

For our planar models, the leg labels are switched at the moment of impact. The hip *q*_*iHip*_, thigh *q*_*iThigh*_ and *q*_*iKnee*_ angles represent leg abduction-adduction, swing and flexion-extension motions, respectively. The thruster angle, *q*_*iThrust*_, represents the rotation around the torso *y*-axis. By considering *p*_1_, we permit the leg-end to bounce and slide on the contact surface.

Leg joints and thruster joints are actuated for a total of eight DOF, while attitude angles and stance leg-end positions constitute the underactuated coordinates, i.e., six DOF. The control vector $u={\left[u_{j}^{⊤},\lambda_{i}^{⊤},F_{1}^{⊤}\right]}^{⊤}$ embodies the joint control actions $u_{j}={\left[u_{iThigh},u_{iKnee},u_{iHip},u_{iThrust}\right]}^{⊤}$ , thruster wrench *λ*_*i*_ and stance leg GRF *F*_1_.

**FIGURE 4:**
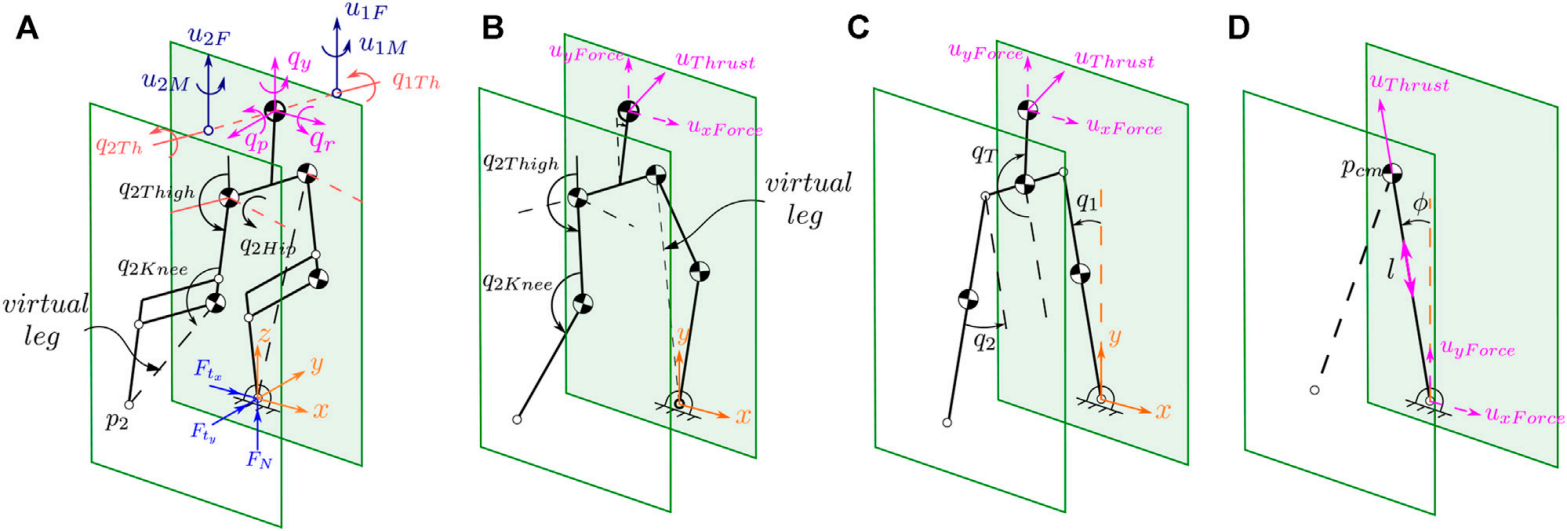
Illustrates the stick diagram of the 3D and 2D models. The full 3D model is shown on the **(A)**. The equivalent five-, three-link and *Variable-Length, Inverted Pendulum (VLIP)* models are shown in **(B–D)**, respectively.

Each co-axial thruster introduces two control actions, including a net thrust force and yaw moment in the thruster coordinate frame. These thruster wrench vectors are mapped (based on Harpy’s geometry) to a net wrench vector in the body coordinate frame $\lambda_{net}={\left[F^{B},M^{B}\right]}^{⊤}$ . This wrench vector consists of the net thruster force and moment on the body frame. This map is given by the adjoint transformation $\lambda_{net}=\sum_{i}Ad_{iB}^{⊤}\lambda_{i}$ , which can be expanded as

$$\lambda_{net}=\overset{2}{\underset{i=1}{\sum }}\left[\begin{array}{cc} R_{i} & 0\\ R_{i}S\left(d_{i}\right) & R_{i} \end{array}\right]\lambda_{i}$$ (1)

where *R*_*i*_ is a rotation about *q*_*iThrust*_, and *S* (*d*_*i*_) is a skew-symmetric matrix that represents the distance component of the cross product ${\vec{d}}_{i}\times F_{i}\;\left(d_{i}\right.$ is the distance from the torso to the thruster). This full-model will be used to validate the control design approach. Also, it will be employed to obtain other low-order models, including five-link, three-link and VLIP models. All of these models can only move in the sagittal plane of locomotion and are shown in Figure 4.

The planar five-link configuration vector embodies seven variables. Torso angle (*q*_*p*_) is measured relative to the vertical line to the ground surface. The thigh and knee angles are defined similarly to the 3D model while the hip joint angles along with the yaw and roll angles about the world coordinate frame are ignored. As a result, only the thruster forces *F*_*xThrust*_ and *F*_*zThrust*_, expressed in the torso coordinate frame, are considered part of the control input. Stance leg-end positions are parameterized with the normal (*p*_*n*_) and tangent (*p*_*t*_) terms to complete a 7-DOF configuration variable vector. The configuration variables for planar three-link and VLIP models are also constructed similarly as shown in Figure 4.

### 4.2 Restricting Frontal and Transversal Dynamics Using Thrusters

The thrusters are located at each side of the body and are aligned with the body COM about the sagittal axis. This design allows the use of the thruster net force and torque around the COM to stabilize the robot’s roll and yaw angles. To restrict the motions of the 3D model to the sagittal plane of locomotion, we employ thruster actions and a simple closed-loop controller. The controller stabilizes the frontal and transversal dynamics. As a result, 2D gaits designed for the sagittal plan of locomotion can be easily applied to the 3D model. For instance, frontal dynamics stabilization is achieved by employing opposite thruster forces at the left and right thrusters.

### 4.3 Single Support Model

At single support, the system retains only a single point of contact with the ground. This contact point is not fixed. A simple static friction model describes the contact force. Regarding the mass allocation in the system, the torso is modeled as a single point mass with no moment of inertia. Since the hip and thigh actuators are located near each other, a single point mass is considered for both of them. Similarly, the knee actuator is modeled as a point mass with no moment of inertia. All of the connecting rods are assumed to be mass-less.

The mass allocation in the planar models is slightly different from the 3D model. For instance, in our five-link model, a single point mass is fixated to the hip joint and is equivalent to the overall mass at the hip in the 3D model. In the three-link model, each leg mass is equivalent to the knee actuator mass, which is fixated to the lower leg in the five-link model. The hip and thigh from the 3D model are summed up and fixated at the hip joint in the three-link model. Last, in the VLIP model, the system’s total mass is considered a point mass.

The governing equations of motion are derived using the methods of Lagrange by taking the kinetic $K\left(q_{s},\dot{q_{s}}\right)$ and potential $V\left(q_{s}\right)$ energies of the system which leads to $L\left(q_{s},\dot{q_{s}}\right)=K\left(q_{s},\dot{q_{s}}\right)-V\left(q_{s}\right)$ . The resulting equations of motion are expressed in vector form as following

$$D_{s}\left(q_{s}\right){\ddot{q}}_{s}+C_{s}\left(q_{s},{\dot{q}}_{s}\right){\dot{q}}_{s}+G_{s}\left(q_{s}\right)=B_{s}\left(q_{s}\right)u,$$ (2)

where *D*_*s*_ (*q*_*s*_) is the symmetric inertial matrix and is only dependent on *q*_*s*_, the $C_{s}\left(q_{s},{\dot{q}}_{s}\right){\dot{q}}_{s}$ matrix contains the Coriolis terms, *G*_*s*_ (*q*_*s*_) contains gravity terms, and the control matrix *B*_*s*_(*q*_*s*_) maps the inputs to the generalized coordinate accelerations ${\ddot{q}}_{s}$ . Consequently, the full-model can be written in a state-space form as

$${\dot{x}}_{s}=\left[\begin{array}{c} {\dot{q}}_{s}\\ D_{s}^{-1}\left(-C_{s}-G_{s}+B_{s}u\right) \end{array}\right]=f_{s}\left(x_{s}\right)+g_{s}\left(x_{s}\right)u,$$ (3)

where the state vector is denoted by $x_{s}={\left[q_{s}^{⊤},{\dot{q}}_{s}^{⊤}\right]}^{⊤}$ . Other planar models are obtained similarly after considering the modeling assumptions explained above. Next, the derivation of the non-instantaneous double support model follows.

### 4.4 Non-Instantaneous Double Support Model

The end of the SS phase is marked by an impulsive impact event when the swing leg-end $p_{2}={\left[p_{tx},p_{ty},p_{n}\right]}^{⊤}$ makes contact with the ground. Then two leg-ends (*p*_1_ and *p*_2_) will be fixed to the ground during the DS phase until the new swing leg lifts off. We will assume fixed DS time intervals for all gait cycles. This switching from the SS to DS phase is captured by defining the switching conditions *p*_2*n*_ = 0 and *p*_2*t*_ > 0. Switching happens when the swing leg-end height, *p*_2*n*_, is zero and its tangential (horizontal) position *p*_2*t*_ > 0, i.e., the swing leg lands in front of the stance leg.

The map to describe leg-end impact is modeled as in the literature Hurmuzlu and Marghitu (1994) which solves for the post-impact states $x_{s}^{+}$ and GRF. The widely employed assumptions of rigid ground models such as instantaneous duration and inelasticity are considered to capture the system’s behavior at the impact moment. As a result, there are no changes in the joints’ position (i.e., $q_{s}^{-}=q_{s}^{+}$ ). Only angular velocity spikes occur at the impact moment. To cover every aspect of thruster-assisted legged locomotion and for the sake of completeness, we will briefly explain this process.

In order to formulate this impact map Δ(*x*_*s*_), the model given by Eq. 3 is considered. The vector *q*_*s*_ is augmented to include the new stance leg-end position *p*_2_, $q_{d}={\left[q_{s}^{⊤},p_{2}^{⊤}\right]}^{⊤}$ . Then, it is assumed that the angular momentum is conserved about the previous stance leg-end *p*_1_ at the moment of impact. The equations of motion are re-formulated to include the impulsive GRF, $\delta J_{e}^{⊤}F_{e}$ , at each leg-end $p_{e}={\left[p_{1}^{⊤},p_{2}^{⊤}\right]}^{⊤}$

$$D_{d}\left(q_{d}\right){\ddot{q}}_{d}+C_{d}\left(q_{d}\right){\dot{q}}_{d}+G_{d}\left(q_{d}\right)=B_{d}\left(q_{d}\right)\left[\begin{array}{c} u_{j}\\ \lambda_{net} \end{array}\right]+\delta J_{e}^{⊤}F_{e}$$ (4)

where $J_{e}={\left[{\left(\partial p_{1}/\partial q_{d}\right)}^{⊤}{\left(\partial p_{2}/\partial q_{d}\right)}^{⊤}\right]}^{⊤}$ and $F_{e}={\left[F_{1}^{⊤},F_{2}^{⊤}\right]}^{⊤}$ . In this equation, $F_{e}\in R^{6}$ is a Lagrange multiplier that assumes both legs are fixed to the ground at the moment of impact. Based on this assumption, we can then formulate this as a zero velocity constraint at the foot-ends, i.e., $\dot{p_{e}}=J_{e}{\dot{q}}_{d}=0$ . Combining this with the conservation of angular momentum, the post-impact states and GRF can be solved as follows

$$\left[\begin{array}{c} {\dot{q}}_{d}^{+}\\ F_{e} \end{array}\right]={\left[\begin{array}{cc} D_{d}\left(q_{d}^{-}\right) & -J_{e}{\left(q_{d}^{-}\right)}^{⊤}\\ J_{e}\left(q_{d}^{-}\right) & 0^{6\times 6} \end{array}\right]}^{-1}\left[\begin{array}{c} D_{d}\left(q_{d}^{-}\right){\dot{q}}_{d}^{-}\\ 0^{6\times 1} \end{array}\right]$$ (5)

The inertial matrix *D*_*d*_ (*q*_*d*_) is a square, symmetric and positive definite, and $J_{e}\left(q_{d}\right)D_{d}{\left(q_{d}\right)}^{-1}J_{e}{\left(q_{d}\right)}^{⊤}$ is full rank Westervelt et al. (2018). As a result, the matrix inversion shown above is possible, which yields the impact map Δ(⋅). After the impact event, both feet stay fixed to the ground. This fixation results in a non-instantaneous DS phase, which is assumed to last for a fixed time interval. This condition is enforced by considering appropriate constraints in the system and is skipped here.

## 5 Control

Here, we outline an overview of our approach to satisfy state, control and GRF constraints during gait cycles by *deforming* the ZD Manifolds whose restriction dynamics governes the gaits. The notion of directional derivatives $L_{f}y=\frac{\partial y}{\partial x}f$ and holonomic constraints *y* = *h*_*s*_ (*x*_*s*_) = *h*_*s*_ (*q*_*s*_) will be adopted in a similar way as mainstream publications in this field.

The holonomic constraints (*y*) are widely known as Virtual Constraints (VCs) since they are enforced by closed-loop feedback Westervelt et al. (2018). Based on these constraints, celebrated control invariant sets of the form $\Gamma =\left\{{\left[q_{s}^{⊤},{\dot{q}}_{s}^{⊤}\right]}^{⊤}\in R^{2n}|G\left(q_{s},{\dot{q}}_{s}\right)=0\right\}$ where $G={\left[h_{s}^{⊤}\left(x_{s}\right),L_{f_{s}}^{⊤}h_{s}\left(x_{s}\right)\right]}^{⊤}\in R^{2\left(n-1\right)}$ and $rank\left\{L_{f_{s}}h_{s}\left(x_{s}\right)\right\}=n-1$ can be defined. The rank condition guarantees that Γ, i.e., $q_{s}=h_{s}^{-1}\left(0\right)$ , is 1-dimensional.

Since there is only one degree of underactuation in all of our planar models, therefore, $h_{s}^{-1}\left(0\right)$ takes the form of a closed curve. We can find a transformation for the configuration variable, $q_{s}^{\prime }=H_{1}q_{s}$ , such that the actuated coordinates are stacked on top the underactuated coordinate. With this in mind, we consider the following parametric descriptions *q*_1_ = *r*_1_ (*q*_*n*_), *…*, *q*_*n*−1_ = *r*_*n*−1_ (*q*_*n*_) where *q*_*n*_ in all of our planar models can be the last entry of *q*_*s*_. As a result, the output function almost similarly takes the following form for all of the models

$$h_{s}\left(q_{s}\right)=H_{2}H_{1}q_{s}-r\left(q_{n}\right)=q_{j}-r\left(q_{n}\right)$$ (6)

Here *q*_*j*_ denotes the actuated joint variables, and *H*_2_ extracts *q*_*j*_ from $q_{s}^{\prime }$ . Note that $r={\left[r_{1},\ldots ,r_{n-1}\right]}^{⊤}$ and the matrix $H_{2}\in R^{\left(n-1\right)\times n}$ can take a trivial form if each joint independently is derived with a single actuator only. The classical problem of enforcing VCs considers fixed gait parameters or, at best, in event-based methods, permits dynamics-free updates of the gait parameters at the boundaries of the gait cycles. As such, by deforming the manifold Γ at the boundaries (i.e., after impact moments), gait characteristics, including average walking speed, step length, etc., are regulated.

However, these methods happen in a discrete-time fashion, and the resulting closed-loop system possesses a very small basin of attraction. Part of the reason the continuous deformation of Γ has never been considered before is that standard bipedal robots, with their small support polygon, cannot achieve stable periodic orbits when $q_{s}=h_{s}^{-1}\left(0\right)$ is deformed. With this observation, we aim to apply the thrusters in two different ways, as discussed earlier in Section 1. In a nutshell, the idea is to continuously deform $q_{s}=h_{s}^{-1}\left(0\right)$ such that the following conditions are satisfied. First, we want $h_{s}^{-1}\left(0\right)$ to remain continuous and form closed curves, i.e. *r* (*q*_*n*_(*t*)) = *r* (*q*_*n*_ (*t* + *T*)), where *T* is the gait period.

Second, we want Γ to remain stabilized at all times; otherwise, enforcing VCs will be impossible. Last, we want gait feasibility conditions, including the equality $C_{eq}\left(q_{n},{\dot{q}}_{n}\right)=0$ and inequality constraints $0\leq C_{ineq}\left(q_{n},{\dot{q}}_{n}\right)$ , to be satisfied. This problem (we refer the readers to Westervelt et al. (2003) for more details) can take the following constrained ordinary differential equation form:

$$\begin{array}{cc} {\ddot{q}}_{n} & =a_{1}\left(q_{n}\right)+a_{2}\left(q_{n}\right){\dot{q}}_{n}^{2}+e\mathrm{ff}ects\;of\;deforming\;h_{s}^{-1}\left(0\right)\\ 0 & \neq \frac{\partial h_{s}}{\partial x_{s}}{\left[0^{⊤},{\left[D_{s}^{-1}\left(q_{n}\right)B_{s}\left(q_{n}\right)\right]}^{⊤}\right]}^{⊤}\;for\;deformed\;h_{\text{s}}^{-1}\left(0\right)\\ q_{n}\left(t+T\right) & =q_{n}\left(t\right)\\ {\dot{q}}_{n}\left(t+T\right) & ={\dot{q}}_{n}\left(t\right)\\ 0 & =C_{eq}\left(q_{n},{\dot{q}}_{n}\right)\\ 0 & \leq C_{ineq}\left(q_{n},{\dot{q}}_{n}\right) \end{array}$$ (7)

where the first line governs the restriction dynamics (*a*_*i*_ (*q*_*n*_) are nonlinear terms) and the second line is the condition for the stabilizability of Γ. Widely considered gait feasibility constraints such as |*x*_*s*_| < *x*_*max*_, |*u*| < *u*_*max*_, 0 < *F*_*N*_ and $|\frac{F_{T}}{F_{N}}|<\mu$ can form the equality and inequality constraints, where *F*_*T*_, *F*_*N*_ are the tangential and normal components of GRF and *μ* is the coefficient of friction.

In our approach, the positive invariance property plays a key role and is closely dependent on how $q_{s}=h_{s}^{-1}\left(0\right)$ is deformed. Positive invariance allows finding the control input *u* such that when *q*_*s*_ (0) is on $q_{s}=h_{s}^{-1}\left(0\right)$ the velocities ${\dot{q}}_{s}\left(t\right)$ remains tangent to $q_{s}=h_{s}^{-1}\left(0\right)$ yielding the solutions *q*_*s*_(*t*) remain on the manifold Γ for all *t* > 0.

If this property is guaranteed, the constraint satisfaction problem can be transformed into a motion planning problem in the state space of the internal dynamics that can be conveniently tackled using simple path-planning tools. This particularly becomes important and handy when fast and reactive gait planning is needed in dynamic walkers. We will further elaborate on this concept with a simple example.

### 5.1 Motivation Behind Valid Deformations of $q_{s}=h_{s}^{-1}\left(0\right)$

To motivate our idea, we will consider $\Sigma :{\left[{\dot{x}}_{1},{\dot{x}}_{2}\right]}^{⊤}={\left[x_{1},x_{2}+u\right]}^{⊤}$ and part of $S^{1}$ set – unit circle in $R^{2}$ – which is $\Gamma =\left\{x_{1},x_{2}|0<x_{1}<1,G\left(x_{1},x_{2}\right)=x_{1}^{2}+x_{2}^{2}-1=0\right\}$ . It is straightforward to show that $u=\frac{-x_{1}^{2}}{x_{2}}-x_{2}$ will stabilize the solutions of Σ, $x\left(t\right)={\left[x_{1}\left(t\right),x_{2}\left(t\right)\right]}^{⊤}$ , on Γ. In this case, the restriction dynamics *f*|_Γ_, which is prescribed by *u*, takes the following form $f{|}_{\Gamma }={\left[x_{1},-x_{1}^{2}/x_{2}\right]}^{⊤}$ . The reason that in our definition of Γ we considered part of $S^{1}$ is evident here, i.e., *x*_2_ ≠ 0.

Now, assume that we have the means to deform Γ and that the goal is to satisfy the constraint given by *x*_1_ ≤ 1/2. The deformation shown in red in Figure 4D cannot result in a stabilizable and consequently positive invariant Γ set. Notice that the control vector field in this example is *g* = [0,1]^*⊤*^ which is shown in Figure 4D. Please note that possible options where $\frac{\partial G}{\partial x}$ and *g*(*x*) are not orthogonal – or *g*(*x*) is transversal to the deformed manifold (Γ) – are not unique.

With this simple example describing the challenge, our first step would be to identify the valid deformations of $q_{s}=h_{s}^{-1}\left(0\right)$ that naturally do not violate the transversality condition (i.e., $\frac{\partial G}{\partial x}⊥̸g\left(x\right)$ ).

### 5.2 Choices and Options on How to Manipulate $h_{s}^{-1}\left(0\right)$

Consider the state-space representation of the system dynamics given by Eq. 3. Here, we will explore our options and choices in order to manipulate Eq. 6. While many options can be considered, we will focus on two general frameworks, as explained below.

With this in mind that the control vector field is given by $g={\left[0^{\left(n-1\right)\times n},\;{\left(D_{s}^{-1}B_{s}\right)}^{⊤}\right]}^{⊤}$ , two possible options are considered: 1) scaling *ω*_*i*_ *q*_*n*_ and 2) shifting *q*_*n*_ + *ω*_*i*_. We are interested in knowing how the stabilizability of *Γ*_*ω*_ is affected if these two options are applied to manipulate the state-dependent equilibrium point of the actuated coordinates. In equilibrium shifting scenario (case ii), notice that the position and velocity priming can be trivially equivalent as is evidently seen below

$$\left[\begin{array}{c} q_{j}-r\left(q_{n}\right)+\omega \left(t\right)\\ {\dot{q}}_{j}-r^{\prime }\left(q_{n}\right){\dot{q}}_{n}+\dot{\omega }\left(t\right) \end{array}\right]\Leftrightarrow \left[\begin{array}{c} q_{j}-r\left(q_{n}\right)+\int_{0}^{T}\nu \left(\tau \right)d\tau \\ {\dot{q}}_{j}-r^{\prime }\left(q_{n}\right){\dot{q}}_{n}+\nu \left(t\right) \end{array}\right]$$ (8)

where $r^{\prime }=\frac{\partial r\left(q_{n}\right)}{\partial q_{n}}$ , *ω*(*t*) and *ν*(*t*) are used for manipulating *h*_*s*_ (*x*_*s*_) and $L_{f_{s}}h_{s}\left(x_{s}\right)$ , respectively. Notice that in our approach the primer variables are time-varying. Constant priming terms yield a discrete collection of parameterized systems, i.e., *x*_*s*_ = *f*_*s*_ (*x*_*s*_) + *g*_*s*_ (*x*_*s*_)*u*_*ω*_, which is not desired here. To see this, it is enough to consider the numerical parameters *ω*, which are used to parameterize *r* (*q*_*n*_, *ω*), as auxiliary control input in discrete Poincare maps. These auxiliary control inputs can only provide discontinuous means of priming *Γ*_*ω*_ at its boundaries.

#### 5.2.1 Stabilizability Condition and First Scenario

We will show that by choosing (i), the continuous manipulation of the primer parameters can violate the transversality condition (*g*_*s*_ (*x*_*s*_)⊥̸*∂h* (*x*_*s*_)/*∂x*_*s*_). To do this, consider *y* = *q*_*j*_ − *r* (*q*_*n*_, *ω*). Then, consider the fact that the inertia matrix *D*_*s*_ (*q*_*n*_) only depends on angles which are evaluated here on *Γ*_*ω*_, i.e., $q_{n}^{\prime }={\left[r^{⊤}\left(q_{n},\omega \right),q_{n}\right]}^{⊤}$ . For a fixed *q*_*n*_, it is possible to show that

$$B_{s}^{*}D_{s}\left(q_{n}\right){\left[r^{\prime }\left(\omega \left(t\right),q_{n}\right),1\right]}^{⊤}$$ (9)

where B* = [0^1×(*n*−1)^, 1] and Eq. 9 can vanish on a point on *Γ*_*ω*_ which implies *g*_*s*_ (*x*_*s*_) and $\frac{\partial h_{s}\left(x_{s}\right)}{\partial x_{s}}$ can be orthogonal. While the orthogonality condition may get violated at only a finite number of points on *Γ*_*ω*_, these violations are not acceptable in our proposed framework and we avoid that. Without loss of generality, consider *M*th order polynomials (e.g., Bezier forms with degree *M* as in ref. [40]) to define

$$r\left(q_{n},\omega \left(t\right)\right)=diag\left(\omega \left(t\right)\right)NQ_{n}$$ (10)

where $diag\left(\omega \left(t\right)\right)\in R^{\left(n-1\right)\times \left(n-1\right)}$ is a matrix with *ω*(*t*) as diagonal entries, $N\in R^{\left(n-1\right)\times \left(M+1\right)}$ is a matrix of constant coefficients and $Q_{n}={\left[1,q_{n},q_{n}^{2},\ldots ,q_{n}^{M}\right]}^{⊤}$ . Then Eq. 9 is re-written in the following simplified form

$$D_{3}\left(q_{n}\right)diag\left(\omega \left(t\right)\right)NQ_{n}^{\prime }+D_{4}\left(q_{n}\right)=0$$ (11)

where *D*_*i*_ (*q*_*n*_) are the block matrices of *D*_*s*_ (*q*_*n*_). This algebraic relationship can be easily solved for a fixed *q*_*n*_ and *ω*(*t*) where $Q_{n}^{\prime }=\partial Q_{n}/\partial q_{n}={\left[0,1,2q_{n},\ldots ,Mq_{n}^{\left(M-1\right)}\right]}^{⊤}$ . Meaning, using this choice of VC priming, it is possible that for some values *ω*(*t*) the transversality condition can be violated on *Γ*_*ω*_.

#### 5.2.2 Stabilizability Condition and Second Scenario

We will now consider the other option, i.e., scaling. It is straightforward to show that $\dot{y}={\dot{q}}_{j}-r^{\prime }\left(q_{n}\right){\dot{q}}_{n}+\omega \left(t\right)$ yields relative degree $\left\{2,\ldots ,2\right\}$ on every points on *Γ*_*ω*_. It is also noticeable that this choice of manipulating *y* has no effects on $null\left\{\frac{\partial h_{s}}{\partial q_{s}}\right\}={\left[r^{\prime ⊤}\left(q_{n}\right),\;1\right]}^{⊤}$ which means that at least the primer has no influence over the transversality condition as long as *y* = *q*_*j*_ − *r* (*q*_*n*_) is a valid VC. Notice that the role of the primer *ω*(*t*) in this form is comparable to the role of a disturbance term in the system given below

$$\left[\begin{array}{c} \dot{y}\\ \ddot{y} \end{array}\right]=\left[\begin{array}{c} {\dot{q}}_{j}-r^{\prime }\left(q_{n}\right){\dot{q}}_{n}\\ L_{f_{s}}^{2}h_{s} \end{array}\right]+\left[\begin{array}{c} 0\\ \left[I,-r^{\prime }\left(q_{n}\right)\right]D_{s}^{-1}\left(q_{s}\right)B_{s} \end{array}\right]u+\left[\begin{array}{c} I\\ 0 \end{array}\right]\omega \left(t\right)$$ (12)

where $L_{f_{s}}^{2}h_{s}=-\left[I,-r^{\prime }\left(q_{n}\right)\right]D_{s}^{-1}\left(q_{s}\right)\left(C_{s}\left(q_{s}\right){\dot{q}}_{s}+G_{s}\left(q_{s}\right)\right)-r^{\prime \prime }\left(q_{n}\right){\dot{q}}_{n}^{2}$ . As a result, the closed-loop system has to possess strong disturbance rejection properties.

The roles of this disturbance term will be beneficial for us, though, as it will adjust the equilibria of *h*_*s*_ (*x*_*s*_) and $L_{f_{s}}h_{s}\left(x_{s}\right)$ under stabilizing controllers with adjustable (and measurable) basins of attraction to successfully satisfy state, control, and GRF constraints. To do this, we need to design an update law for the primer variable vector *ω*(*t*) such that the finite-time convergence of the solutions to *Γ*_*ω*_ in the closed-loop system is unaffected. In addition, while the evolution of the trajectories in the constraint-admissible subspace (this will be explained shortly) of the state space is secured, we want *q*_*j*_ closely track predefined *r* (*q*_*n*_, *ω*(*t*)) when possible. While stronger stability properties (e.g., global exponential stability) are desirable, our major concern will be the finite-time enforcement of the changed virtual constraints.

As far as the design of *u* is concerned, any nonlinear controller (or linear controllers if the nonlinear terms are bounded and the bounds are known) can satisfy the VCs. We will limit ourselves to the following modest feedback law $u=-{\left(L_{g}L_{f}h_{s}\left(x_{s}\right)\right)}^{-1}\left(L_{f}^{2}h_{s}\left(x_{s}\right)+K_{P}y+K_{D}\dot{y}\right)$ where $K_{i}\in R^{\left(n-1\right)\times \left(n-1\right)}$ are constant matrices and instead will remain focused on deforming *Γ*_*ω*_ in order to satisfy our constraints. Hence, we will assume stabilizing supervisory controllers that guarantee the enforcement of the virtual constraints. However, their disturbance rejection properties have to be carefully considered.

The control law given above can generate Global Asymptotic Stability (GAS) at the equilibrium point of the system given by Eq. 12 when the primer variable vector is time-invariant, i.e., *ω*(*t*) = *ω*. Subsequently, *Γ*_*ω*_ takes the following form

$$\Gamma_{\omega }=\left\{x_{s}\in X|h_{s}\left(x_{s}\right)=K_{P}^{-1}K_{D}\omega ,L_{f_{s}}h_{s}\left(x_{s}\right)=-\omega \right\}$$ (13)

where the equilibrium points for *h*_*s*_ (*x*_*s*_) and $L_{f_{s}}h_{s}\left(x_{s}\right)$ under *ω* are obtained by solving

$${\left[\begin{array}{c} h_{s}\left(x_{s}\right)\\ L_{f_{s}}h_{s}\left(x_{s}\right) \end{array}\right]}_{\omega }=\left[\begin{array}{cc} K_{P}^{-1}K_{D} & K_{P}^{-1}\\ -I & 0 \end{array}\right]\left[\begin{array}{c} \omega \\ 0 \end{array}\right]$$ (14)

Of course, realizing GAS property under *ω*(*t*), i.e., when the primer variable is time-varying, as we shall see later, will not be achieved trivially and requires a regulated rate of change in the primer variable vector. We will discuss this in the proof of GAS property of the closed-loop system later in Section 5.4.

A traditional approach to deal with a time-varying disturbance is to synthesize a family of linear controllers at each equilibrium point of the system given by Eq. 12 and then synthesize the stabilizing controller based on interpolating between the linear controllers by gain scheduling. However, nonlinear update laws will produce better results. Using them, it would be possible to directly incorporate the constraints, particularly when gait re-planning has to happen quickly.

Next, with the role of the primer variable *ω*(*t*) in our output function *y* = *h*_*s*_ (*q*_*s*_, *ω*(*t*)) set, we will show that states, joint torques and GRF can be expressed in terms of the primer variable.

### 5.3 Constraints Derivation

Consider the configuration variable vector $q_{s}^{\prime }=H_{1}q_{s}={\left[q_{j}^{⊤},p_{1}^{⊤},q_{n}\right]}^{⊤}$ where $q_{j}\in R^{m}$ are actuated joint angles, *m* is the number of these joints in the planar models and *p*_1_ is stance leg contact point as defined previously. The control matrix in the Euler-Lagrange equations can take the following form

$$B_{s}=\left[\begin{array}{ccc} 0 & 0 & {\left(\frac{\partial p_{T,COM}}{\partial q_{j}}\right)}^{⊤}\\ I^{m\times m} & 0 & {\left(\frac{\partial p_{T,COM}}{\partial p_{1}}\right)}^{⊤}\\ 0 & I^{2\times 2} & {\left(\frac{\partial p_{T,COM}}{\partial q_{n}}\right)}^{⊤} \end{array}\right]$$ (15)

where *p*_*T*,*COM*_ is the physical location of the thruster action *F* ^*B*^. Notice that based on how the thruster actions *F* ^*B*^ are incorporated in the Euler-Lagrange equations (Eq. 2), the coordinate *q*_*n*_, which was primarily assumed to be underactuated, will be actuated. While this is not a problem, to keep our desired normal form – i.e., *q*_*n*_ remains underactuated – and to allow the direct regulation of GRF, *F* ^*B*^ is found such that $F^{B}\in null\left\{\frac{\partial p_{T,COM}}{\partial q_{n}}\right\}$ . As result, the models shown in Figure 4 will be equivalent and the thruster forces *F* ^*B*^ can be translated to the base of the VLIP model based on the law of translating forces and moments.

In other words, if it is assumed that $\tan\left(q_{n}\right)=\frac{F_{x}^{B}}{F_{z}^{B}}$ , the GRF components $F_{x}^{B}$ and $F_{z}^{B}$ can be shifted to the leg-end point. This leaves us with an underactuated *q*_*n*_ angle as it was planned and the possibility of directly regulating GRF. Note that aligning *λ*_*net*_ with the virtual leg in the model takes place by employing the thruster joint actuators *F* ^*B*^ which are responsible to move the thrusters with respect to the body.

One interesting interpretations of the orthogonality condition (i.e., $F^{B}\in null\left\{\frac{\partial p_{T,COM}}{\partial q_{n}}\right\}$ ) is that thruster-assisted legged locomotion over any surface with friction coefficients as small as near zero values becomes feasible. However, the major challenge, when walking over a very slippery surface, i.e., $\tan\left(q_{n}\right)=\frac{F_{x}^{B}}{F_{z}^{B}}=\frac{F_{T}}{F_{N}}<<1$ , is that smaller COM swing motions (i.e., Δ*q*_*n*_) are allowed which can be problematic given that in our design framework all joint motions are parameterized based on *q*_*n*_. In other words, very small Δ*q*_*n*_ means no locomotion.

Based on the adjustment we made in the control matrix *B*_*s*_ and that $q_{s}=h_{s}^{-1}\left(0\right)$ is stabilizable, for the previously defined $B_{s}^{*}$ the following will hold $B_{s}^{*}D_{s}\left(q_{n}\right){\left[r^{\prime ⊤}\left(\omega \left(t\right),q_{n}\right),1\right]}^{⊤}=\alpha$ where *α* ≠ 0. As a result, the following equation can be obtained at every point on *Γ*_*ω*_ and it governs the dynamics of the ${\left[q_{n},{\dot{q}}_{n}\right]}^{⊤}$ curve

$${\ddot{q}}_{n}=-\alpha^{-1}\left(\beta_{1}{\left({\dot{q}}_{n}\right)}^{2}+\beta_{2}\right)$$ (16)

In this equation $\beta_{2}=B_{s}^{*}G_{s}\left(q_{n}\right)$ and *β*_1_ is given by

$$\beta_{1}=B_{s}^{*}\left(D_{s}\left(q_{n}\right){\left[r^{\prime \prime ⊤},0\right]}^{⊤}+\Sigma_{i=1}^{n}\left[r^{\prime ⊤},1\right]Q_{i}\left(q_{n}\right){\left[r^{\prime ⊤},1\right]}^{⊤}\right)$$ (17)

where *Q*_*i*_ (*q*_*n*_) are the Christoffel Symbols. A similar algebraic relationship for the constraints ${\left[u_{j}^{⊤},\lambda_{net}^{⊤}\right]}^{⊤}$ is obtained. Following the relationships given by Eqs. 6 and 8, constraints equations can be written in terms of *y* and $\dot{y}$ , which is skipped here to avoid a cluttered notation.

Next, we will steer *y* and $\dot{y}$ using the primer variable *ω*(*t*) in Eq. 12 in order to make sure the solutions of Eq. 16 stay within the constraint-admissible space. To do this, consider *y*- $\dot{y}$ space. While a similar analysis is possible in the *q*_*n*_- ${\dot{q}}_{n}$ space, the *y*- $\dot{y}$ space can offer some geometric tools that can help formulate the problem as a classical motion planning problem. Obviously, the result of such adjustments would be the deviation from the nominal gait trajectories, which can affect, e.g., hybrid invariance. We will use the thrusters’ actions to deal with these issues.

Since we assumed a pre-stabilized system – in fact all of the above derivations only make sense if $q_{j}=r\left(q_{n}\right)+\int_{0}^{t}\omega \left(\tau \right)d\tau$ and ${\dot{q}}_{j}=r^{\prime }\left(q_{n}\right){\dot{q}}_{n}+\omega \left(t\right)$ – it is reasonable to evaluate the constraints $c_{l}\leq {\left[u_{j}^{⊤},F_{1}^{⊤}\right]}^{⊤}\leq c_{u}$ (*c*_*l*_ and *c*_*u*_ are constraint lower and upper bounds) based on the steady-state solutions, i.e., *y*_*ω*_ and ${\dot{y}}_{\omega }$ , and ignore the transient solutions, i.e., ${\left[y^{⊤},{\dot{y}}^{⊤}\right]}^{⊤}=e^{At}z_{0}+\int_{0}^{t}e^{A\left(t-\tau \right)}B_{\omega }w\left(\tau \right)d\tau$ , where $z_{0}={\left[y_{0}^{⊤},{\dot{y}}_{0}^{⊤}\right]}^{⊤}$ is the initial condition and

$$A=\left[\begin{array}{cc} 0^{\left(n-1\right)\times \left(n-1\right)} & I^{\left(n-1\right)\times \left(n-1\right)}\\ -K_{P} & -K_{D} \end{array}\right]$$ (18)

This assumption may cause the intermittent violation of the constraints, which is expected. However, because finite-time convergence to the constraint-admissible sub-spaces at the neighborhood of *y*_*ω*_ and ${\dot{y}}_{\omega }$ is guaranteed – currently we assume that this holds true and later we ensure the GAS property is achieved – by the GAS property of the controller, any transient constraint violations are quickly compensated.

Other than simplifying the nonlinear constraint satisfaction problem given in Eq. 7, considering ${\left[y_{\omega }^{⊤},{\dot{y}}_{\omega }^{⊤}\right]}^{⊤}$ has another interesting result which will be explained below. Consider the set

$$Y_{\omega }=\left\{z\in R^{2\left(n-1\right)}|\left[I^{\left(n-1\right)\times \left(n-1\right)},-K_{P}^{-1}K_{D}\right]z=0\right\}$$ (19)

which is the locus of all of the steady-state solutions of the system Eq. 12. We will show that it is possible to create positive invariant sets around any point $z_{\omega }={\left[y_{\omega }^{⊤},{\dot{y}}_{\omega }^{⊤}\right]}^{⊤}$ in the set defined by *Y*_*ω*_. Then, one can slide ${\left[y_{\omega }^{⊤},{\dot{y}}_{\omega }^{⊤}\right]}^{⊤}$ over the hyperplane defined by *Y*_*ω*_ using a suitable update policy to ensure the following objectives are achieved.

First objective is the realization of positive invariance property in a neighborhood around the steady-state solutions on *Y*_*ω*_. Second objective is to satisfy gait feasibility constraints as outlined before. Last, we want to maintain a minimal distance from the origin, i.e., $min\left\{\left\|{\left[y_{\omega }^{⊤},{\dot{y}}_{\omega }^{⊤}\right]}^{⊤}-0\right\|\right\}$ . The last condition implies that the modified gaits should closely match the predefined gaits. The nominal (predefined) gait parameters are obtained off-line without taking into account the constraint equations given by Eq. 7.

### 5.4 Primer Variable Vector, *ω*(*t*), Update Policy and Achieving Global Asymptotic Stability Property Over *Y*_*ω*_

**FIGURE 5:**
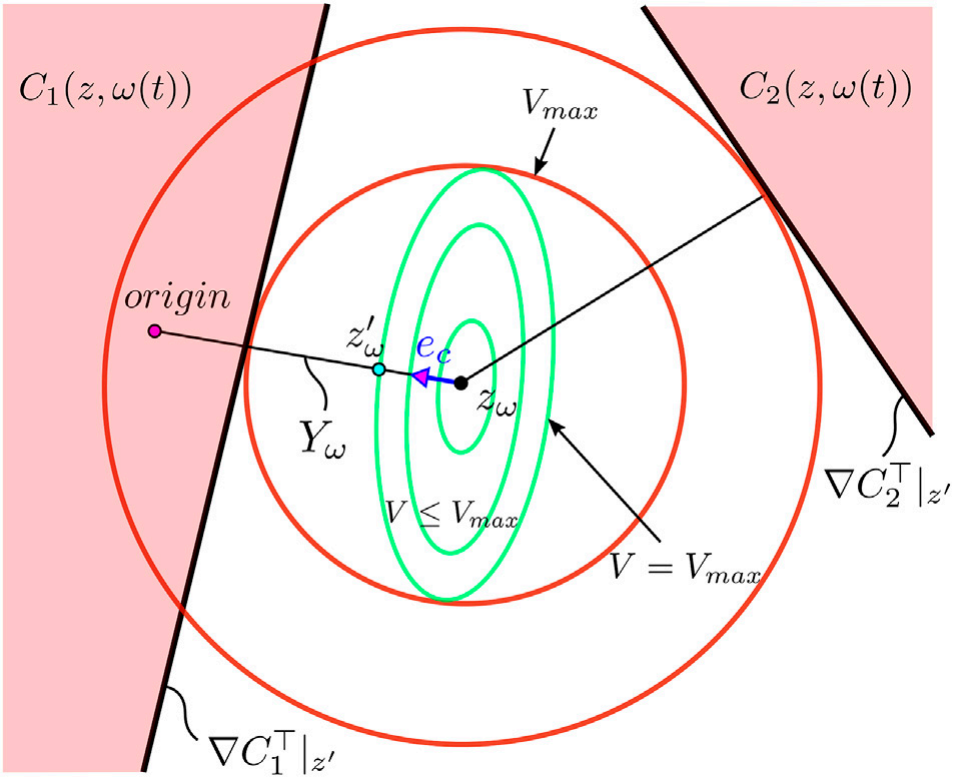
Geometric interpretation of the level set {*ζ*_*w*_|*V* ≤ *V*_*max*_}. For a constant reference *r*, the update law $\dot{w}$ to the manipulated reference *w*, places the new value w* at the edge of the level set *V* = *V*_*max*_ while trying to be as close to the actual reference as possible without violating the constrains *C*_*i*_(*z*, *ω*(*t*)), which are depicted in red.

**FIGURE 6:**
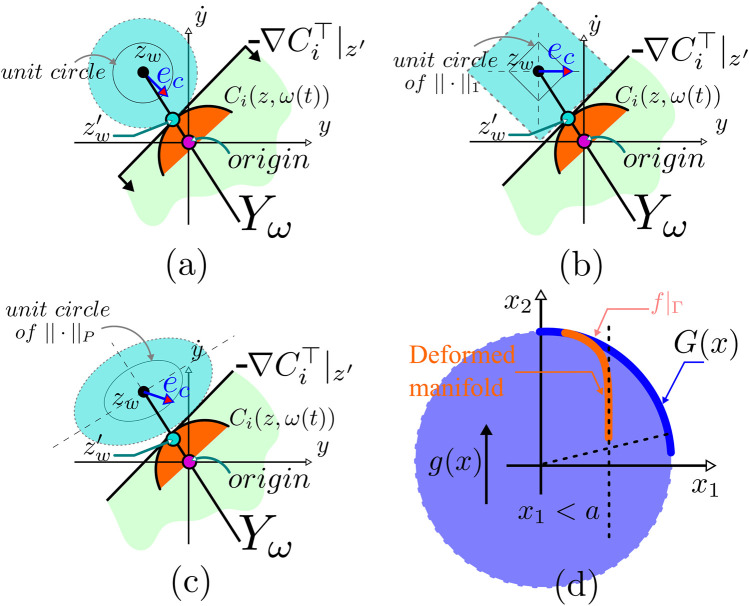
**(A–C)** Illustration of constraint satisfaction based on manifold deformation; Also, it shows the concept with various norm definitions. **(D)** Illustration of the relationship between the stabilizability of the manifold Γ (unit circle is used as an example) and the transversality condition, i.e., $\frac{\partial G}{\partial x}⊥̸g\left(x\right)$ at any point on Γ; *G*(*x*) is the algebraic equation that defines Γ and *g*(*x*) is the control vector field used to stabilize the trajectories on Γ.

Consider the support hyperplane given by

$$c_{i}={\left(\nabla C_{i}\left(z,\omega \left(t\right)\right){|}_{z_{\omega }^{\prime }}\right)}^{⊤}\left(z-z_{\omega }^{\prime }\right)=0$$ (20)

at the point $z_{\omega }^{\prime }$ where the nonlinear constraint *C*_*i*_(*z*, *ω*(*t*)) – i.e., the *i*th entry in the constraint vector *C* (*z*, *ω*(*t*)) given by Eq. 7 – and *Y*_*ω*_ intersect (see Figures 5, 6). Since there are multiple constraints, we consider the constraint with the shortest distance from *z*_*ω*_ on *Y*_*ω*_. While there are many ways to simplify the geometric representation of the nonlinear constraints *C*_*i*_(*z*, *ω*(*t*)) at the neighborhood of *z*_*ω*_ on *Y*_*ω*_, the approach adopted here is very efficient with a minimum computation overhead.

Our approach is motivated by classical methods widely used in constraint optimization problems based on support hyperplanes. These methods are usually very conservative, but in our case their use is justified as the system is highly nonlinear, unpredictable. That said, the location and distance of these hyperplanes from *z*_*ω*_ has to be carefully defined.

We will use Lyapunov functions with quadratic forms to define level sets around *z*_*ω*_. The largest invariant set confined in the constraint-admissible space will define the maximum distance between *z*_*ω*_ and *c*_*i*_ (*z*, *ω*(*t*)). These invariant sets, geometrically, can take various forms using non-quadratic energy functions. It is important to note that the geometry of the invariant set around *z*_*ω*_ directly affects the performance of this approach, i.e., the convergence rate to $z_{\omega }^{⊤}=0$ and constraint error margins (shown in Figure 6).

Motivated by deepest gradient descent method which is a key mechanism of manipulating pre-compensated systems, we consider two search parameters including a step direction, *ρ* = − *ω*(*t*)/‖*ω*(*t*)‖_2_, ‖*ω*(*t*)‖_2_ ≠ 0, and step length, $\delta \in R^{+}$ .

The step direction *ρ* is trivially obtained based on the observation that the primer variable *ω*(*t*) and consequently the steady-state solutions *z*_*ω*_ should remain possibly very close to the origin. The step length *δ* is obtained based on the difference between the values of a candidate Lyapunov functions and the largest invariant sets around *z*_*ω*_. Based on these two search parameters, the following update law for the primer variable vector *ω*(*t*) is considered:

$$\dot{\omega }\left(t\right)=k\rho \delta$$ (21)

where $k\in R^{+}$ is the tuning parameter.

Consider *ζ* = *P* ^−0.5^ *z* and *ζ*_*ω*_ = *P* ^−0.5^ *z*_*ω*_ where *P* is a positive definite matrix. The maximum level set value *V*_*max*,*i*_ (*ω*(*t*)) for the quadratic Lyapunov function $V\left(\zeta ,\zeta_{\omega }\right)=V\left(\zeta ,\omega \left(t\right)\right)={\left(\zeta -\zeta_{\omega }\right)}^{⊤}P\left(\zeta -\zeta_{\omega }\right)$ is obtained by doing the change of variable given above. In this way, *V*_*max*,*i*_ (*ω*(*t*)) can geometrically be interpreted with unit circles around *z*_*ω*_ in *z*-space. The size of the largest level set *V*_*max*,*i*_ (*ω*(*t*)) that is not violating the closest constraint $c_{i}\left(z,\omega \left(t\right)\right)=c_{i,1}^{⊤}z+c_{i,2}$ to the point *z*_*ω*_ can be better imagined when 2-norm ‖.‖_2_ is used, shown in Figure 6. The unit vector $e_{c_{i}}$ along the shortest distance between *z*_*ω*_ and the constraint hyperplane *c*_*i*_ = 0 is given by $e_{c}=\nabla C_{i}{|}_{z_{\omega }}/\|\nabla C_{i}{|}_{z_{\omega }}{\|}_{2}$ and the largest level set value is given by $V_{max,i}\left(\omega \left(t\right)\right)=c_{i,2}/\sqrt{c_{i,1}^{⊤}c_{i,1}}$ .

Now consider the quadratic norm ‖*ζ*‖_*P*_. Notice that using the change of variable *ζ* = *P* ^−0.5^ *z* in the constraint equation *c*_*i*_ (*z*, *ω*(*t*)) = 0, 2-norm and the quadratic norm become interchangeable. In this way, the maximum level set value for any candidate, quadratic Lyapunov function can be obtained. Therefore, the step length in Eq. 21 is given by *δ* = *V*_*max*,*i*_ (*ω*(*t*)) − *V* (*ζ*, *ω*(*t*)). After the change of variable, the unit vector $e_{c_{i}}$ which now is the maximum distance traveled towards the constraint hyperplane from *ζ*_*ω*_ in a unit ball of ‖.‖_*P*_ is given by

$$e_{c_{i}}=-{\left(\gamma^{⊤}P^{-1}\gamma \right)}^{-0.5}P^{-1}\gamma$$ (22)

where $\gamma =\nabla C_{i}{|}_{z_{\omega }}$ . To obtain the equation, we considered the dual norm of the quadratic norm ‖*γ*‖_*p*_ which is ‖*γ*‖_*_ = ‖*P* ^−0.5^ *γ*‖_2_ and this algebraic relationship $e_{c_{i}}=-P^{-1}\gamma /\|\gamma {\|}_{*}$ .

Now, we make sure under the update law given by Eq. 21 the constraint-admissible set defined at any point on *Y*_*ω*_ is a positive invariant set. Taking any Lyapunov function of the form given above and using Barbashin-Krasovskii-LaSalle principle, for a constant primer variable *ω* the GAS property of the closed-loop system given by Eq. 12 is readily achievable.

Take the time-varying primer variable *ω*(*t*) governed by the update law given above, then $\dot{V}\left(\zeta ,\omega \left(t\right)\right)$ is given by

$$\dot{V}\left(\zeta ,\omega \left(t\right)\right)=-{\left(\zeta -\zeta_{\omega }\right)}^{⊤}Q\left(\zeta -\zeta_{\omega }\right)+2{\left(\zeta -\zeta_{\omega }\right)}^{⊤}P{\dot{\zeta }}_{\omega }$$ (23)

where *Q* is a positive definite matrix. For $\dot{\omega }\left(t\right)\neq 0$ , there exists 0 < *κ* ≪ 1 such that

$$\|2{\left(\zeta -\zeta_{\omega }\right)}^{⊤}P^{0.5}A^{-1}B_{\omega }\frac{\dot{\omega }\left(t\right)}{\kappa }\|\leq \lambda_{\min}\left(Q\right)\|\zeta -\zeta_{\omega }{\|}^{2}$$ (24)

As a result, the resulting inequality $\dot{V}\left(u,\omega \left(t\right)\right)<\left(\kappa -1\right)\lambda_{\min}\left(Q\right)\|z{\|}^{2}<0$ holds because it is assumed *κ* ≪ 1. This result shows that if the primer dynamics is very fast, i.e., 1/*κ* in Eq. 21 is a very large value, then the closed-loop system given by Eq. 23 is GAS.

Next, we briefly show how the thruster can be used to achieve hybrid invariance.

### 5.5 Thruster-Assisted Impact Invariance

One critical aspect of HZD-based methods is selecting *y* = *h*_*S*_ (*x*_*s*_) that leads to a hybrid zero dynamics. In these methods, the assumption of underactuation at the contact points does not leave any options for control better than the deadbeat hybrid extension of these systems to achieve impact invariance. This section briefly discusses another use of the thruster actions to secure hybrid invariance, complementary to HZD-based methods.

At the transition from SS to DS phase, the two-point impact, as discussed in Section 4.4, renders all of the body joints except the torso joint fixated to their pre-impact values. Hence, large deviations in joint velocities from the reference trajectories and subsequently from the ZD manifold (*Γ*_*ω*_) will be resulted.

Since the joint actuators are not able to make corrections needed to steer the states back to *Γ*_*ω*_ the thrusters can be leveraged in the DS phase to achieve hybrid invariance. Here, impact invariance such that the initial DS phase state *x*_*d*,0_ returns to the initial SS phase state *x*_*s*,0_ is sought where $x_{d}={\left[q_{d}^{⊤},{\dot{q}}_{d}^{⊤}\right]}^{⊤}$ .

As opposed to the SS phase, the constraints in the DS phase take a more complex form. Additionally, we have to ensure that the final state of the DS phase (*x*_*d*,*f*_) matches the initial states at the SS phase (*x*_*s*,0_).

We apply a Nonlinear Model Predictive Control (NMPC) scheme to steer the post-DS states back to the ZD manifold. This scheme is known for being costly. However, the duration of the DS phase is significantly shorter than the SS phase. Note that a reference trajectory for each DS state *r*_*d*_ [*k*] is generated at every *k*th sample over the duration of the DS phase. The reference trajectory can be a simple linear trajectory between the post-impact states *x*_*d*,0_ and the initial SS phase state *x*_*s*,0_.

The continuous DS phase model as described in Eq. 4, along with the kinematic constraint $J_{d}\ddot{q}+{\dot{J}}_{d}\dot{q}=0$ results in the following differential algebraic equation

$$\left[\begin{array}{cc} D_{d}\left(q_{d}\right) & -J{\left(q_{d}\right)}^{T}\\ J_{d}\left(q_{d}\right) & 0^{\left(6\times 6\right)} \end{array}\right]\left[\begin{array}{c} {\ddot{q}}_{d}\\ F_{e} \end{array}\right]=\left[\begin{array}{c} B_{d}\eta -H_{d}\left(q_{d},{\dot{q}}_{d}\right)\\ -{\dot{J}}_{d}\left(q_{d},{\dot{q}}_{d}\right) \end{array}\right]$$ (25)

where *η* = [*u* ^*⊤*^, *λ*_*net*_] is an augmented input vector that consists of the control action from the SS phase along with the net thrust force along the torso, which can be expressed in the state-space form given by ${\dot{x}}_{d}=f_{d}\left(x_{d}\right)+g_{d}\left(x_{d}\right)\eta$ . This model is discretized at each sample time. To cast this as an NMPC problem a cost function *ϕ*(*x*_*d*_, *η*) to be minimized is formulated resulting in the following optimization problem

$$\begin{array}{cc} \underset{\eta \left[k\right]}{min}\;\phi \left(x_{d},\eta \right) & =\overset{N}{\underset{k=1}{\sum }}W_{r}\|\left|\right.\left|\right.\left(x_{d}\left[k\right]-r_{d}\left[k\right]\right)\left|\right.{\left|\right.}_{2}^{2}+W_{\eta }\left|\right.\left|\right.\delta \eta \left[k\right]\left|\right.{\left|\right.}_{2}^{2}\\ subj.to:\\ & C_{eq}\left(x_{d},\eta \right)=0\\ & C_{ineq}\left(x_{d},\eta \right)=0 \end{array}$$ (26)

In Eq. 26, *W*_*r*_ is the weighting term for the cost associated with tracking the reference trajectory *r*_*d*_ [*k*], *W*_*η*_ similarly contains the weight that penalizes rapid changes *δ* in the input *η*, and *N* denotes the total simulation time steps. The constraints of the optimization problem are denoted by *C*_*eq*_ (*x*_*d*_, *η*), *C*_*ineq*_ (*x*_*d*_, *η*) which represent the equality and inequality constraints, respectively. The equality terms include the initial DS state, which is obtained from the post-impact SS state $x_{s}^{+}$ , and the discretized dynamics ${\dot{x}}_{d}$ .

$$C_{eq}\left(x_{d},\eta \right):\left\{\begin{array}{cc} x_{d}\left[0\right]\; & =R_{s}^{d}x_{s}^{+}\\ x_{d}\left[k+1\right]\; & =f\left(x_{d}\left[k\right]\right)+g\left(x_{d}\left[k\right]\right)\eta \left[k\right] \end{array}\right.$$ (27)

In Eq. 27, the matrix $R_{s}^{d}$ swaps the roles of the legs. The inequality equations include the difference between the final DS state and initial SS state, GRF constraints and the limits imposed on the state and input vectors.

$$C_{ineq}\left(x_{d},\eta \right):\left\{\begin{array}{cc} \left|x_{d}\left[N\right]-x_{s}\left[0\right]\right| & \leq \epsilon \\ \left|\right.|F_{T}\left[k\right]|\left|\right. & <\mu \left|\right.{|F}_{N}\left[k\right]|\left|\right.\\ 0 & <{|F}_{N}\left[k\right]|\\ \left|\right.{|x}_{d}\left[k\right]\left|\right.| & <x_{d\;max}\\ \left|\right.|\eta \left[k\right]\left|\right.| & <\eta_{max} \end{array}\right.$$ (28)

Here *ϵ* is a relaxation term applied to the final state. With all constraints satisfied, the NMPC guides the DS states towards the initial condition of SS phase, resulting in impact invariance.

## 6 Simulation Results

**FIGURE 7:**
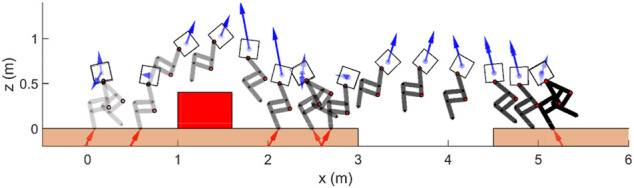
An overview of Harpy’s controller architecture.

In this section, we will report the result of our simulation works. An overview of the control architecture explained previously is given in Figure 7. We consider walking on flat ground when Harpy makes point contacts with the surface. Using the full-dynamics model explained above, we will apply the thruster actions and proposed framework to demonstrate the desired capabilities, including, frontal stabilization, trajectory manipulation to stay in constraint-admissible regions of the state space, hybrid invariance and high jumps over obstacles.

Since one of the motivations behind our approach is to reduce computation overhead, we will briefly demonstrate the results from integrating our ROMs (i.e., the VLIP, 3-link, etc., models explained previously) in this process. We will demonstrate that this integration can further reduce the costs.

We note that the gaits are designed based on the HZD approach. In the SS phases, the desired trajectories *h*_*d*_ (*x*_*s*_) are parameterized as Bezier polynomials with the coefficients tuned offline. All model parameters used in the simulation closely match Harpy’s properties and are listed in Table 1.

**TABLE 1:** Model parameters.

Parameter	Value	Description
*m*_*T*_	300 *g*	Mass of torso
*m*_*h*_	200 *g*	Mass of hip
*m*_*k*_	100 *g*	Mass of each leg
*l*_*T*_	30 *cm*	Length from hip to torso
*l*_1/2*Thigh*_	18 *cm*	Length from hip to knee
*l*_1/2*Tibia*_	32 *cm*	Length of tibia
*l*_1/2*Meta*_	32 *cm*	Length of metatarsus
*l*_1/2_	63.25 *cm*	Length of leg on three link ROM

**FIGURE 8:**
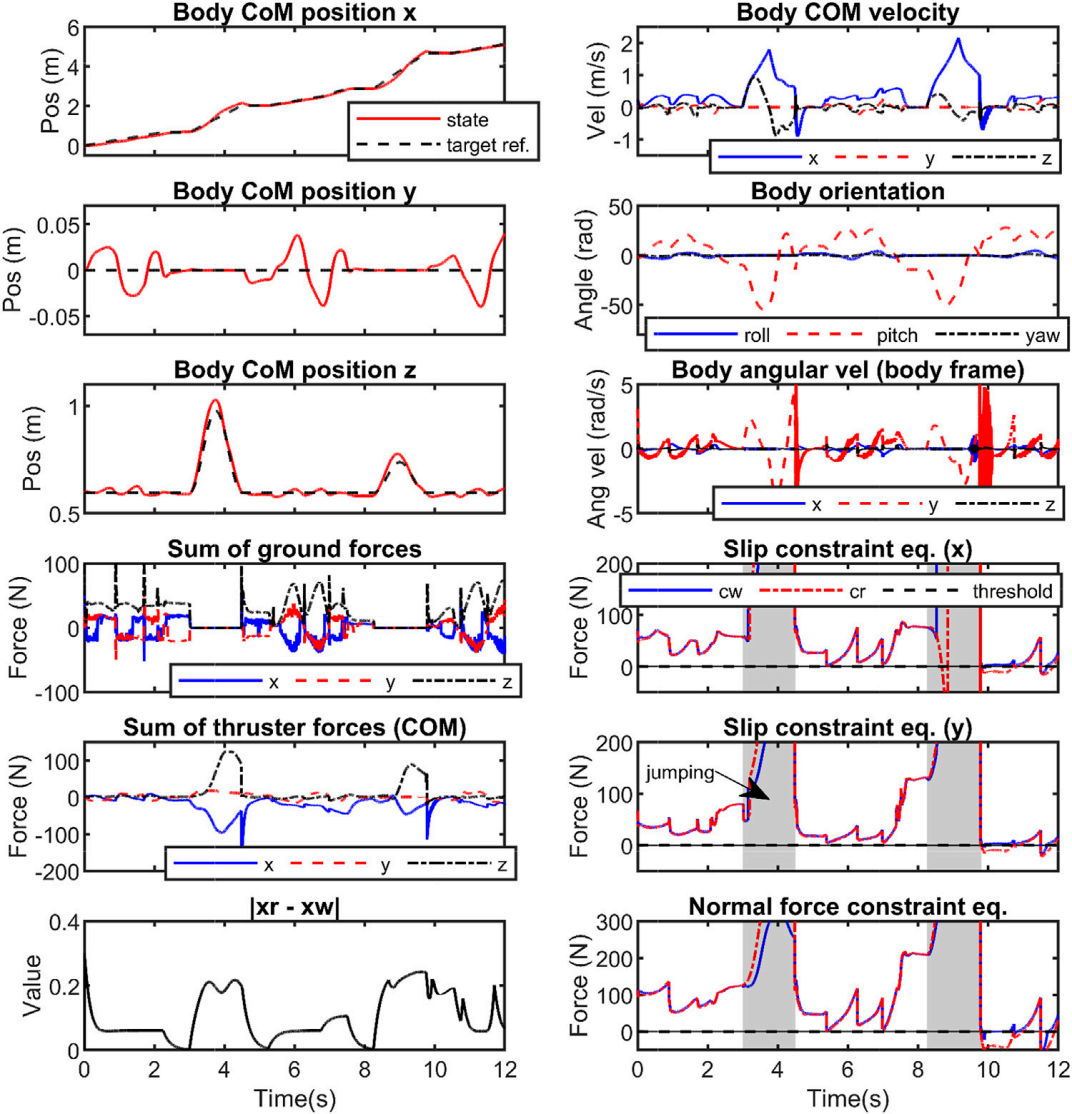
Illustration of thruster-assisted locomotion over a rough terrain simulated on Harpy’s full-dynamics. Blue and red arrows show the thruster actions and GRF, respectively.

The full-dynamics model can be constrained in its frontal plan of locomotion using the thrusters. We note that frontal dynamics stabilization due to underactuation can be very challenging. We ran the simulations using the full-dynamics model of Harpy where the legs are modeled with all DOF including the hip frontal, hip sagittal, and knee sagittal joints. Figure 8 shows the stick-diagram results from several key states during walking. The thrusters not only are used to stabilize the frontal dynamics, but also to jump over obstacles, and assist the robot to land stably.

**FIGURE 9:**
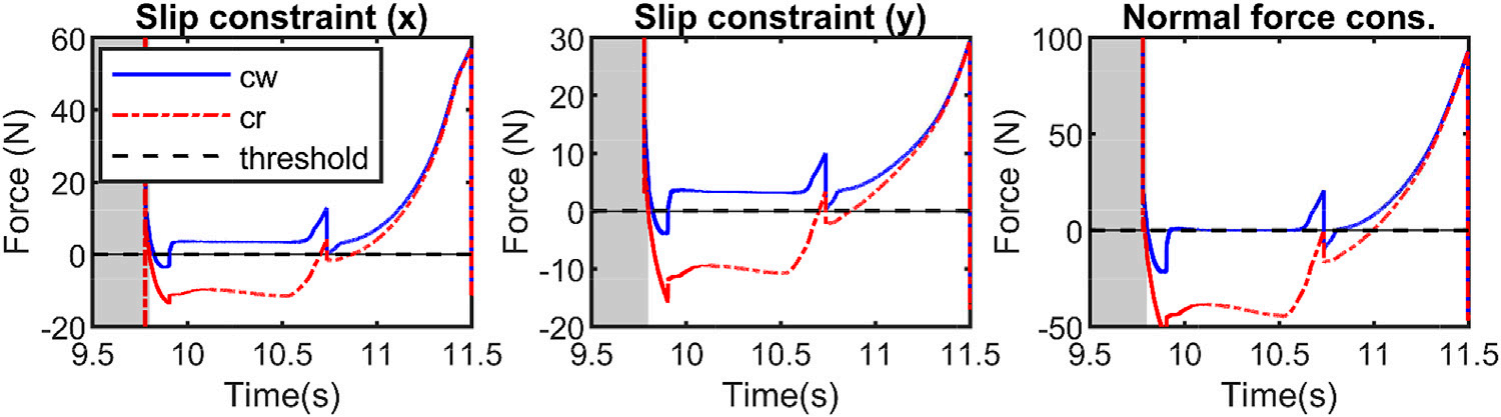
Illustrates the state values and forces during walking and jumping feats simulated with Harpy’s 3D full-dynamics. The reference joint trajectories (*z*_0_) are manipulated (*z*_*ω*_) such that the applied constraint equations do not violate the constraint as shown at *t* = [9.5, 11.5]s.

The simulation results such as the robot’s states and GRF are shown in Figure 9. In general, planar gaits are unstable in 3D systems. The simulation result demonstrate that the thruster-assisted robot is capable of tracking the target body trajectory and walk stably even when the 2D gait is used. The thrusters can assist the robot to walk with a stable and nearly zero roll and heading angles throughout the simulation and can be seen in Figure 8.

**FIGURE 10:**
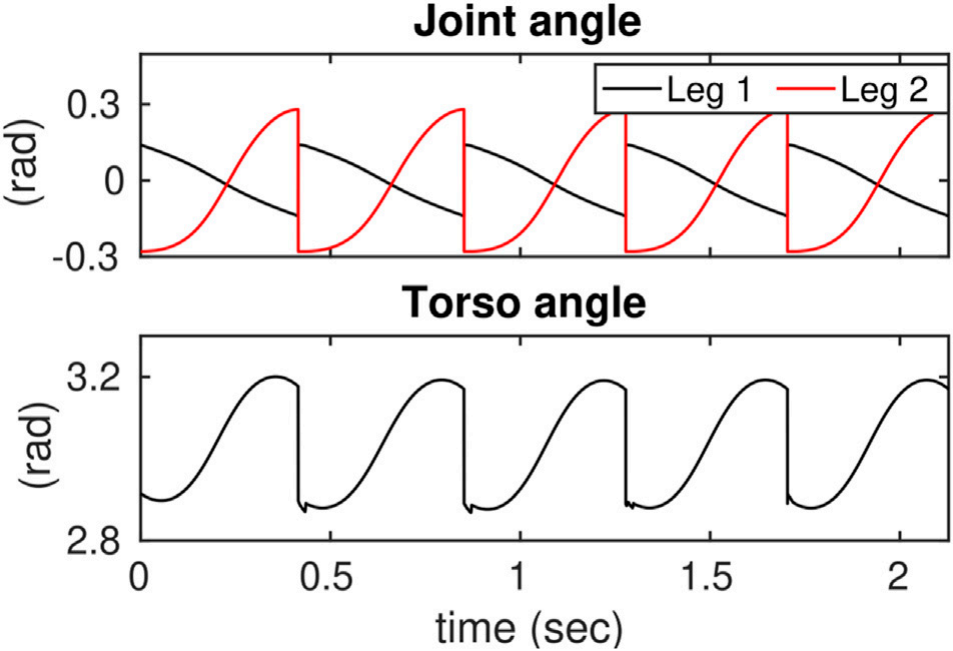
Illustrates the effects of reducing the control thresholds on the control actions and their corresponding phase portraits.

**FIGURE 11:**
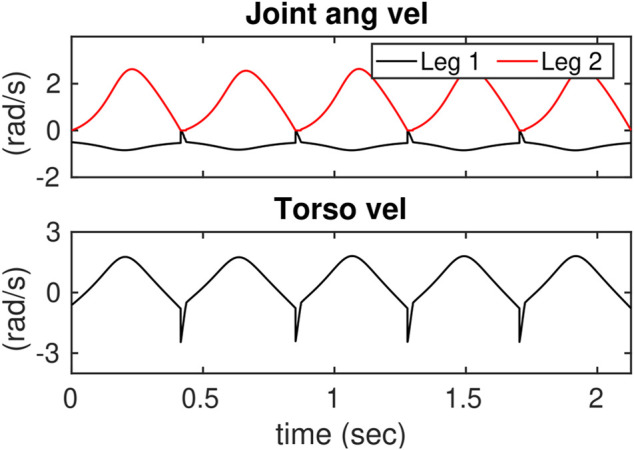
Illustrates the closeup view of the constraint violations during 3D walking and jumping at *t* = [9.5, 11.5]s. The reference trajectories violate the constraints and the controller manipulates the applied references to prevent the constraints from being violated.

In Figures 9, 10, the thrusters are used to satisfy the constraints including saturating the control actions and enforcing no-slip conditions. First, a desired trajectory for the COM of the full-dynamics is considered and using the priming approach explained above it is manipulated during the walking phase to satisfy GRF feasibility constraints. The primer is disabled during the flight phase. As shown in Figure 9, the GRF constraints are violated at around *t* = [9.5, 11.5]. Figure 11 depicts a close-up view of the priming performance at *t* = [9.5, 11.5].

**FIGURE 12:**
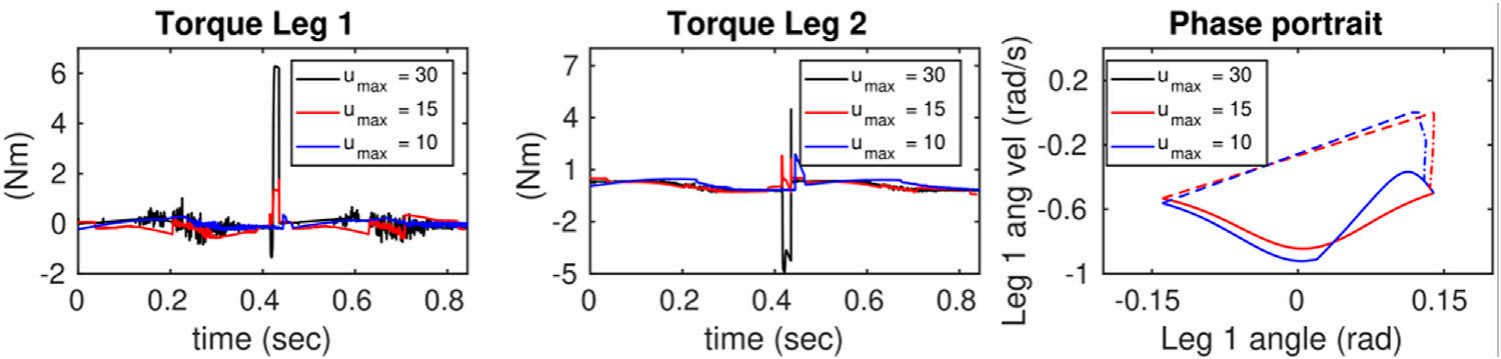
Illustrates the reference joint angle trajectories in the 3-link ROM.

**FIGURE 13:**
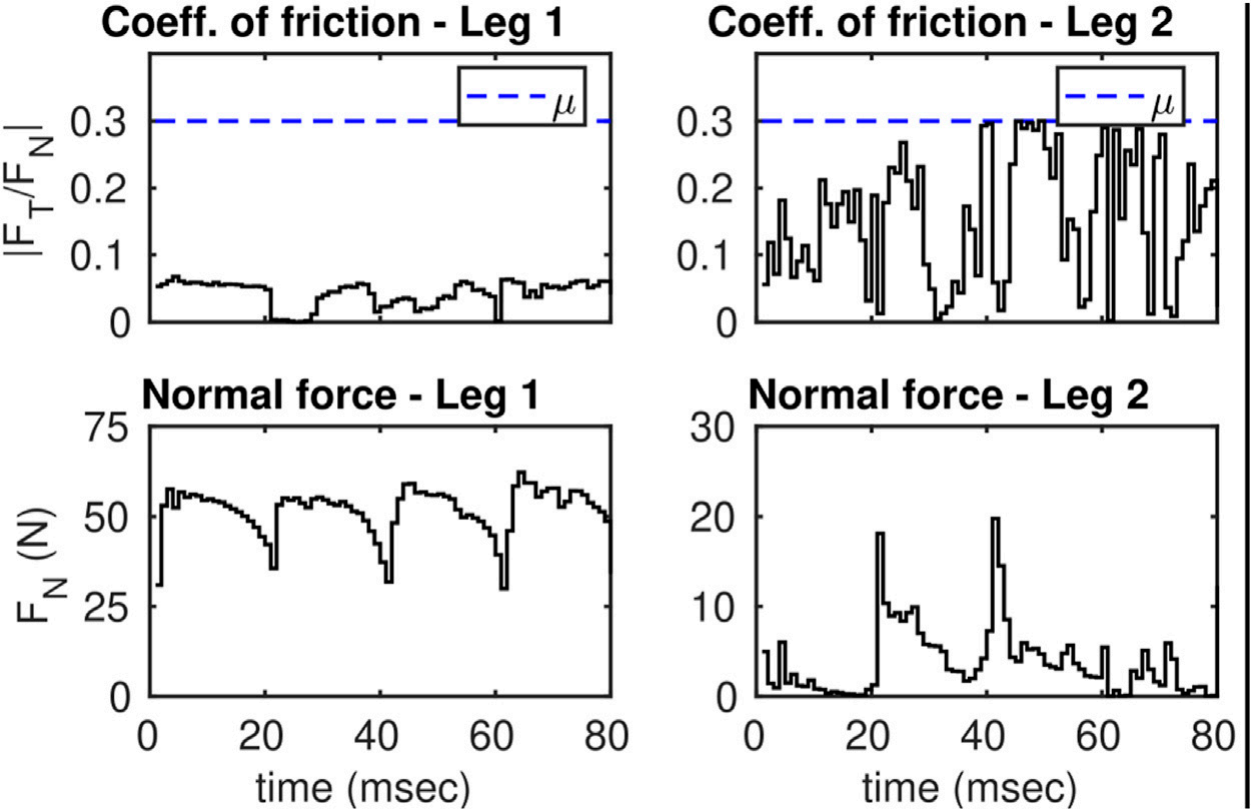
Illustrates the reference joint velocity trajectories in the 3-link ROM.

In addition, we considered constraining the control actions. To do this, the control inputs were saturated in the low-dimensional space of the ROMs and then were mapped to the full-dynamics. The priming problem to saturate control inputs in the three-link ROM is considered. Figures 12, 13 show the desired joint angles and velocities, respectively.

In Figure 10, the results after applying the primer are shown. The first two figures from left show the effects of changing the limits on the control inputs during the SS phase. The limits are adjusted from high to low values resulting in the peak control to decrease below the desired thresholds. However, a large gap can be seen between the threshold and maximum control input. Note that the geometries of the positive invariant sets around steady-state responses in the output dynamics are not optimal in our work as explained before and shown in Figure 5. The phase portraits **(** ***q***_**1**_ **-** ${\dot{q}}_{1}$ **)** corresponding to each control limits are shown in Figure 10. It can be seen that $h_{s}^{-1}\left(0\right)=q_{s}$ is deformed to satisfy the constraints which can affect hybrid invariance in the locomotion system.

**FIGURE 14:**
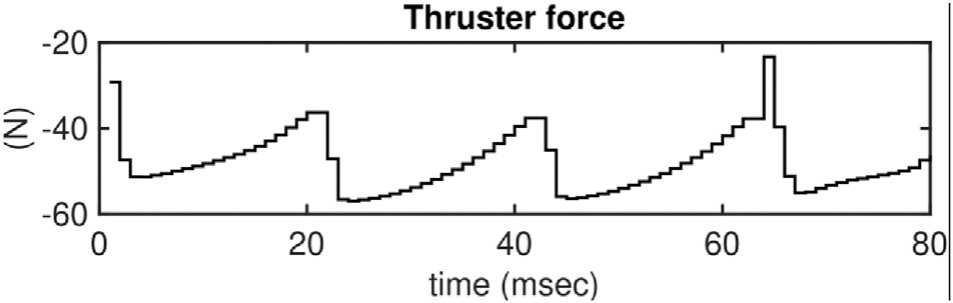
Illustrates the friction constraints during the DS phase of leg 1 and 2. In this plot, the intermediate SS phases are omitted. Dashed blue lines are the thresholds.

Hybrid invariance can be achieved using the thruster actions. Figure 14 shows the feasibility conditions during the DS phase where NMPC is applied. The friction cone condition is feasible which indicates that both feet are fixated to the ground throughout the DS phase.

**FIGURE 15:**
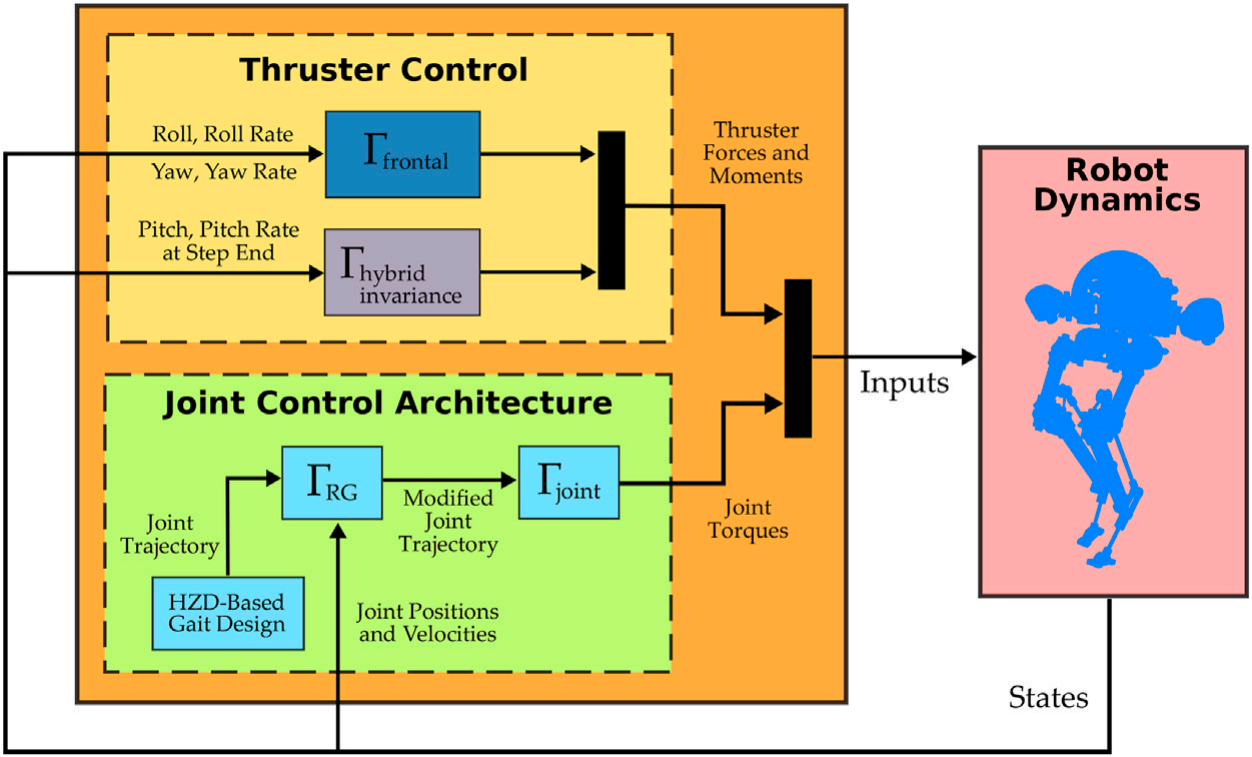
Illustrates the thruster actions during the DS phase. In this plot, the intermediate SS phases are omitted as the thrusters are inactive.

Note that the static friction coefficient is assumed to be *μ* = 0.3. Also note that the normal forces spike to 60 N during the DS phase. The total weight of the biped is only 0.8 kg which suggests that the unusual behavior is only possible because of the thruster actions. Figure 15 shows the control actions for the thrusters during the DS phase.

## 7 Concluding Remarks

In this work, we studied the roles of thrusters in addressing the prevailing challenges in bipedal robotics, potentially paving the way to see new designs with unexplored capabilities. Some of the common challenges faced by bipedal robots include constraint satisfaction, frontal dynamics stabilization, and avoiding fallovers. Combatting these issues can be pretty challenging in these systems due to underactuation and high vulnerability to external perturbations.

In this publication, we introduced a thruster-assisted bipedal robot called Harpy. Harpy platform possesses two legs and two coaxial thrusters attached to its torso. We capitalized on Harpy’s unique design to propose an optimization-free approach to satisfy Harpy’s gait feasibility conditions, including control and contact forces. The reference trajectories were manipulated based on deforming stabilizable zero-dynamics manifolds to fulfill constraints brought on by ground contacts and those prescribed by states and inputs without violating hybrid invariance.

In standard bipedal robots, unintended changes to the restriction dynamics over the zero dynamics manifold, especially those optimized to produce periodic orbits, can adversely affect gait stability. Not only we showed that the manipulation of the system trajectories is possible in Harpy full-dynamics, but also hybrid invariance can be realized by employing the thrusters, something that is often achieved in a limited fashion by event-based regulators.

In addition, we demonstrated that the thrusters can be utilized to robustify the gaits by dodging fallovers or even jumping over large obstacles.

## Data Availability

The raw data supporting the conclusions of this article will be made available by the authors, without undue reservation.
